# RNA and neuronal function: the importance of post-transcriptional regulation

**DOI:** 10.1093/oons/kvac011

**Published:** 2022-07-07

**Authors:** Vandita D Bhat, Jagannath Jayaraj, Kavita Babu

**Affiliations:** Centre for Neuroscience, Indian Institute of Science, CV Raman Road, Bangalore 560012, Karnataka, India; Centre for Neuroscience, Indian Institute of Science, CV Raman Road, Bangalore 560012, Karnataka, India; Centre for Neuroscience, Indian Institute of Science, CV Raman Road, Bangalore 560012, Karnataka, India

**Keywords:** RNA editing and neuronal granules, alternative splicing

## Abstract

The brain represents an organ with a particularly high diversity of genes that undergo post-transcriptional gene regulation through multiple mechanisms that affect RNA metabolism and, consequently, brain function. This vast regulatory process in the brain allows for a tight spatiotemporal control over protein expression, a necessary factor due to the unique morphologies of neurons. The numerous mechanisms of post-transcriptional regulation or translational control of gene expression in the brain include alternative splicing, RNA editing, mRNA stability and transport. A large number of *trans*-elements such as RNA-binding proteins and micro RNAs bind to specific *cis*-elements on transcripts to dictate the fate of mRNAs including its stability, localization, activation and degradation. Several *trans*-elements are exemplary regulators of translation, employing multiple cofactors and regulatory machinery so as to influence mRNA fate. Networks of regulatory *trans*-elements exert control over key neuronal processes such as neurogenesis, synaptic transmission and plasticity. Perturbations in these networks may directly or indirectly cause neuropsychiatric and neurodegenerative disorders. We will be reviewing multiple mechanisms of gene regulation by *trans*-elements occurring specifically in neurons.

## INTRODUCTION

Post-transcriptional regulation (PTR) of mRNA is thought to be essential at every step of its complex life cycle as it travels from the nucleus to the cytoplasm. In neurons, the influences of *trans*-elements, such as RNA-binding proteins (RBPs) and non-coding RNAs, provide an essential means through which most complex molecular pathways are regulated post-transcriptionally. *Trans*-elements function via their ability to recognize specific *cis*-elements such as sequences or structures present on both precursor and mature mRNAs. They serve as important gatekeepers of gene expression in multiple processes such as splicing, translation, RNA transport, RNA stability and decay that ultimately dictate several aspects of neuronal functioning, including cognition.

Neurons are known for their extraordinary anatomical and functional diversity. While this remarkable phenomenon has long been recognized, little is known about how this diversity arises. It is achieved, in part, through highly complex translational control that dictates RNA metabolism. For instance, pre-mRNA processing events such as alternative splicing (AS) and RNA editing contribute greatly to the proteome diversity and functional complexity of the nervous system.

There are many indicators that allude to the role of *trans*-element facilitated PTR in establishing neuronal diversity. These include long genes coding for signaling molecules, receptors, ion channels and cell adhesion molecules that are essential for neuronal communication and connectivity. The disrupted forms of these genes are implicated in autism spectrum disorders (ASDs) and Rett syndrome [82, 201, 230]. Long neuronal effector genes have been suggested to contribute disproportionately to neuronal transcriptional diversity [200, 253]. This hypothesis is bolstered by studies where long genes were shown to be expressed at higher levels in neurons when compared to non-neuronal cells in the nervous system [82, 201]. Moreover, long genes tend to have larger numbers of exons and therefore are ideal targets for post-transcriptional regulatory mechanisms such as AS, which results in the expression of large numbers of distinct isoforms. Additionally, the untranslated regions (UTRs) of mRNA are a hotbed of *cis*-elements for RBPs and non-coding RNA (ncRNA), particularly the 3′-UTR. Brain tissues were observed to express higher levels of mRNA isoforms with long 3′-UTR as opposed to short 3′-UTRs [42, 173, 248]. Of the several cell types in the brain, longer 3′-UTR isoforms were particularly enriched in neurons as opposed to astrocytes, microglia and oligodendrocytes [226, 239, 244].

**Figure 1:**
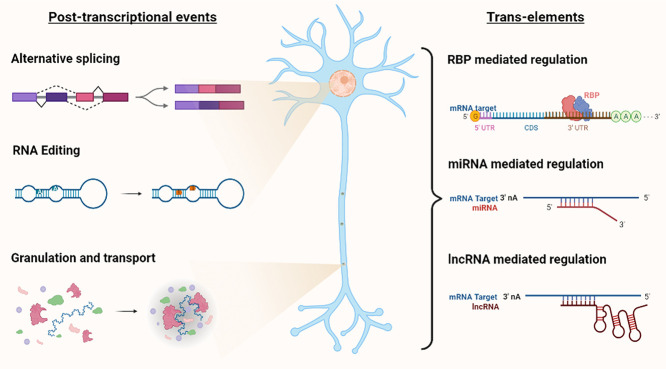
Illustration depicting key post-transcriptional events and *trans*-elements involved in involved in PTR in neurons.

Thus, it is essential to understand how regulatory factors function whether it be individually or in conjunction with other factors. This review attempts to condense their roles their mechanism of action through which they achieve translational control during several processes. While these means of regulation are also rampant in non-neuronal cells, we have focused on neurons for the sake of brevity. We have also chosen to place emphasis on specific examples that are typical of regulation by *trans*-elements that involve one-on-one interactions with their target mRNAs leading to changes in gene expression and synaptic plasticity. This article will focus on reviewing multiple processes of gene regulation occurring in neurons and will highlight some of the underlying mechanisms that allow for neuronal gene expression and function (illustrated in Fig. 1).

## AS AND NEURONAL DIVERSITY

AS is essential for RNA processing during several critical stages of neuronal development and function—memory consolidation, plasticity, cell signaling neuronal differentiation, axon guidance and synapse formation (reviewed in [80, 131, 132]). AS has the capacity to generate hundreds to several thousands of splice variants in the nervous systems of animals [213]. AS can affect downstream regulatory processes such as nonsense-mediated decay (NMD) and add additional layers of post-transcriptional gene regulation ([124] and reviewed in [99, 119]). Most of these splicing events cause inclusion or exclusion of conserved protein domains. They may also introduce a frame shift or a premature stop codon, which triggers NMD. The series of events that lead to NMD could potentially regulate transcript stability, diversity and abundance in the transcriptome of different neuronal cell populations ([212, 242] and reviewed in [4]). Regulated splicing is seemingly coupled to NMD that ultimately determines protein abundance via the regulation of the transcriptome, i.e. isoform diversity and transcript abundance ([124, 242] and reviewed in [143]).

AS of pre-mRNAs produces functionally distinct isoforms that are essential for neuronal development, plasticity, complex behaviors and cognition (reviewed in [132]). AS is a common mechanism to diversify genetic output in metazoans and is especially prevalent in the mammalian nervous system (reviewed in [24, 142]). Neuronal splicing events expand the mammalian neuronal transcriptome and are regulated by several *trans*-factors such as RBPs and microRNAs (miRNAs). Aberrant splicing has been implicated in several neurodegenerative disorders and splicing-correcting therapies have been successfully used to improve disease symptoms [77, 84, 97, 195]. Thus, understanding the underlying mechanisms of AS regulation by *trans*-elements is critical for discovering new therapeutic interventions.

*Trans*-factors that regulate splicing have been demonstrated to be expressed in a tissue-specific manner to influence AS forms of particular transcripts. They control the choice of splicing within a transcript by binding to the pre-mRNA and enhancing or silencing specific splicing events [24, 142]. The *trans*-factors discussed in this review are enlisted in Table 1. AS alters exon composition by generating numerous exon combinations in mRNA isoforms, which are then translated into the final proteins. Coordinated spatiotemporal AS regulation by RBPs is an important contributor to neuronal diversity. RBP families including PTB, RBFOX, NOVA, serine/arginine-rich, STAR and heterogeneous nuclear ribonucleoproteins (hnRNPs) that bind to RNA motifs to regulate AS. RBPs determine alternative exon usage by recognizing *cis*-regulatory elements on pre-mRNAs ([223] and reviewed in [80]).

**Table 1:** List of *trans*-factors discussed in this review

***Trans*-factor/s**	**Category of *trans*-factor**	**mRNA target**	**Function**	**Significance**
PTB	RBP	*mef2*	AS	Maintaining survival and neuronal function in N2A cells [92, 252]
miR-124	miRNA	*ptb*	mRNA degradation	Degradation of PTB in neurons upregulates nPTB expression, which is crucial for nervous system development [136]
SLM2	RBP	*nrxn1,2* and *3*	AS	Specification of glutamatergic synapse [213]
RBFOX	RBP	*ankG*	AS	Formation of axon initiation segment [104]
		*trkb*	Translational control	Promotes BDNF dependent long-term memory [210]
		*vamp*	Translational upregulation	Aids Inhibitory synaptic transmission during learning and memory [222]
FBF-1	RBP	*egl-4*	Translational upregulation and localization	Adaptation to odors, i.e. olfactory memory [108]
ADR-2	RBP	*clec-41*	RNA editing leading to stability and translational upregulation	Regulates chemotaxis in *C. elegans* [50]
TDP-43	RBP	*cdkn1a*	Translational upregulation	Induces cell cycle block leading to cell death [221]
HUD	RBP	*nrn1*	Localization of Nrn1 mRNA in axons	Promotes synapse formation [147]
		*gap-43*	Axonal localization	Assist axon growth and regeneration [150]
Translin	RBP	*bdnf*	Dendritic localization	LTP and synaptic plasticity [165]
FMRP miR-125b	RBP miRNA	*nr2a*	Maintains NMDA receptor ratio by marking mRNA for degradation	Promotes synaptic plasticity [64]
BACE1-AS	lncRNA	*bace1*	Stability and translational upregulation	Aberrant cleavage of APP leading to Alzheimer’s disease [69]
miR-485-5p	miRNA	*bace1*	MRNA degradation	Prevents APP aggregation by degrading the BACE1 enzyme that aberrantly cleaves APP [69]

### AS is highly context-dependent and neuron-specific

**Figure 2:**
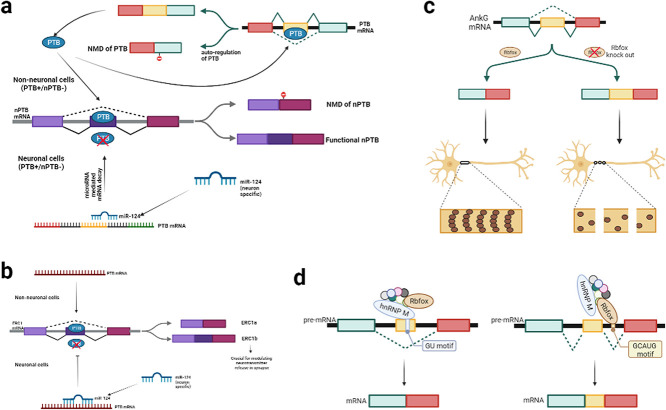
(a) Neuron specific splicing patterns of PTB and nPTB. In non-neuronal cells, PTB mRNA undergoes normal translation forming functional PTB that in turn binds to nPTB mRNA leading to exon skipping and NMD. Also, PTB binds to its own mRNA in a concentration dependent manner, resulting in NMD, thus ensuring homeostasis. However, in neuronal cells, miR-124 is expressed that binds to the PTB mRNA leading to translational suppression. Lower concentration of PTB restricts its binding to nPTB mRNA (adapted from [43, 96]). (b) Similar to nPTB, PTB mediates the AS pattern of ERC mRNA. Presence of PTB in non-neuronal cells leads to exon exclusion in ERC mRNA leading to synthesis of ERC1a isoform, whereas in non-neuronal cells, ERC1b isoform is expressed, which contains an additional exon (adapted from [26]). (c) RbFox determines the splicing pattern of AnkG mRNA. Knockout of *RbFox* leads to exon inclusion in AnkG resulting in a mutant protein that impairs assembly of the AIS (adapted from [104]). (d) RbFox with hnRNP M and other regulators forms the large macromolecular assembly of splicing regulator complex that acts constitutively based on the presence of corresponding binding sites and is involved in changes in splicing patterns (adapted from [46]).

AS can be highly context-dependent and neuron-specific (reviewed in [80]). During neural development, some AS events are reprogrammed by *trans*-factors causing switching in protein expression. RBPs are known to bind pre-mRNA and control AS by binding sequence-specific sites. AS may be controlled by multiple RBPs depending on the cell identity. Different *trans*-factors bind to the same RNA target sequence but may create distinct splicing outcomes in different tissues or developmental stages [223]. An apt example of this is PTB and neural PTB (nPTB) RBPs that are discussed below and illustrated in Fig. 2a and b.

Polypyrimidine tract-binding proteins, PTB and nPTB are structurally similar paralogs across all four of their RNA recognition motifs (RRMs) and well-known AS regulators [139, 164]. They bind to CU-rich regulatory regions near PTB-repressed exons and alter the assembly of the spliceosomes at adjacent splice sites [2, 128, 164, 194]. While PTB is expressed in neuronal precursor cells, glia and other non-neuronal cells, nPTB expression is restricted to post-mitotic neurons and is actively repressed in non-neuronal cells in part due to PTB-induced AS of nPTB mRNA, leading to NMD. This mutually exclusive pattern of expression of PTB and nPTB in the brain during neuronal differentiation is established post-transcriptionally and controls numerous splicing programs (reviewed in [198, 224]).

### Post-transcriptional reprogramming of AS

The switch in RBP alters the splicing of many alternative exons that may be included or excluded in neuronal mRNA. PTB and nPTB target genes are essential for the remodeling of neurons during axon outgrowth and synaptogenesis ([26, 45, 251] and reviewed in [51, 93]). nPTB also interacts with the neuron-specific splicing regulator and RBP, NOVA1 [176]. PTB, and not nPTB, strongly represses the alternative β exon of transcription factor MEF2 (MEF2 plays a key role in neuronal function and survival) in N2A cells during P19 differentiation into neurons ([138, 252] and reviewed in [92, 192]). Rab6IP2 protein (also called ERC1), which is expressed in two spliced isoforms, is involved in membrane trafficking. ERC1a is ubiquitously expressed, but ERC1b is neuron-specific due to inclusion of an exon and modulates neurotransmitter release at the synapse. ERC1b neuron-specific exon is repressed by PTB in non-neuronal cells. Loss of PTB function leads to the synthesis of synaptic ERC1b due to AS, implicating this event in the synthesis of components required for efficient presynaptic function during neuronal differentiation [227]. This is illustrated in Fig. 2b.

The K homology (KH)-domain of RBP, SLM2, is essential for the correct specification of glutamatergic synapses in the mouse hippocampus. SLM2 modifies the AS of endogenous neurexin in the hippocampus. Genome-wide mapping has revealed highly selective SLM2-dependent AS events consisting of very few mRNAs that encode synaptic proteins including the neurexins (Nrxn1, 2 and 3). Neurexins are presynaptic transmembrane cell-adhesion molecules that play a significant role in synapse regulation. The neurexin mRNA has six splice sites, of which splice site 4 is extensively studied. The inclusion or exclusion of this splice site determines the ligand to which neurexin binds. SLM-2 mediates the AS of neurexin-1 at splice site 4 [44].

Genetic change to a single SLM2-dependent target exon in Neurexin-1 rescued synaptic function, plasticity and behavioral defects in *Slm2* knockout mice. SLM2 is selectively expressed in specific neuronal cell types and regulates very few alternative exons that ultimately dictate synaptic properties in neuronal circuits [213].

### Rbfox family of RBPs and AS

Rbfox family of RBPs consists of three highly conserved splicing factors, Rbfox1 (A2bp1), Rbfox2 (Rbm9) and Rbfox3 (NeuN), that contribute to neuronal development and maturation ([104] and reviewed in [114]). Studies in mouse models have demonstrated the tissue specificity of these Rbfox splicing factors, i.e. RBFOX1 is specific to the brain, the heart and the muscle, whereas RBFOX2 is expressed in multiple organs like the ovary, the brain, the kidney, the heart, the lung epithelium, the muscle and the embryo. Comparatively, RBFOX3 is not well characterized and generally found in neurons [33].

RBFOX1 regulates a wide array of AS networks during neuronal development. Defects in RBFOX1 have been implicated in many neurodevelopmental and neuropsychiatric disorders including ASD, bipolar disorder, schizophrenia, attention-deficit hyperactivity disorder and epilepsy (reviewed in [23]). RNA sequencing performed to study RBFOX1 splicing network in primary human neural stem cells during differentiation implicates it in the AS events of genes involved in key developmental processes such as cellular proliferation, cytoskeletal organization, cell adhesion and signaling. Thus, RBFOX1 may behave as a molecular switch that promotes correct differentiation of neuronal progenitors into different functional cell types [78]. The role of RBFOX in splicing is illustrated in Fig. 2c and d.

Rbfox proteins regulate AS by binding to conserved (U)GCAUG elements in the transcript [107, 127, 177]. While strong binding to canonical Rbfox motifs is evident, several additional motif variants or secondary motifs were also detected in abundance in previous studies. Rbfox proteins bind to the secondary motifs *in vivo* and their regulatory function with respect to these motifs is dependent on Rbfox concentration, which is especially high during neuronal development. The Rbfox concentration-dependent regulation of splicing events adds an additional layer of control during neuronal differentiation and cell type specification [20]. Although Rbfox proteins recognize highly conserved binding sites on target mRNA, they do not function in isolation. Their structures and diverse roles suggest that they interact with various protein partners to achieve several outcomes. Nuclear Rbfox proteins are part of a large macromolecular assembly of splicing regulators (illustrated in Fig. 2d). Rbfox are bound within this multimeric complex that also contains non-RRM domain proteins such as hnRNPs like hnRNP M, hnRNP H, hnRNP C, nuclear matrix protein (Matrin3), double-stranded RBP (NF110/NFAR-2), RBP with domain associated with zinc fingers (NF45) and DEAD-box helicase (DDX5). This defined set of splicing cofactors affects Rbfox by enhancing its target recruitment considerably, thereby demonstrating additional complexities of splicing networks [46].

Loss of function alleles of *Rbfox1* and *Rbfox2* makes mice more susceptible to seizures and causes cerebellar defects and ataxia [85, 86]. Mutations in *Rbfox* genes are also associated with autism, schizophrenia and epilepsy [14, 21, 140, 190, 240]. *Rbfox* triple knockout ventral spinal neurons have defects in AS of cytoskeletal, membrane and synaptic proteins and go on to display aberrant electrophysiological activity [104].

Rbfox depletion causes the defective assembly of the axon initial segment (AIS) that is a subcellular structure important for action potential initiation and consequently regulates neuronal excitability and plasticity (reviewed in [181]). Maturation of AIS relies on the recruitment and accumulation of AnkG in the proximal axon that is responsible for assembling essential binding partners [91]. AIS defects observed in *Rbfox* triple knockout neurons are caused by defective splicing of AnkG that is unable to integrate into the actin cytoskeletal lattice. Exon 34 of AnkG codes for a short peptide that inhibit AnkG-βIV spectrin interaction, where βIV spectrin plays a critical role in linking ankG/Na^+^ channel to actin cytoskeleton (reviewed in [101]). AnkG function requires exclusion of an alternative exon mediated by Rbfox, which in turn leads to the proper establishment of AIS and, subsequently, neuronal maturation [104]. This is illustrated in Fig. 2c.

### Coordination of RBPS and miRNAS during AS events

RBPs are also known to work in conjunction with the action of miRNAs to control AS. In the case of PTB, previously mapped binding sites are situated close to predicted miRNA target sites [241]. This bolsters the possibility of RNA stability regulation by PTB via functional interplay with miRNAs.

Multiple neuron-specific miRNA, such as miR-9/9* and miR-124, play important roles in neuronal differentiation [245]. miR-9 post-transcriptionally inactivates the PTB paralog *nPTB* by targeting its binding site in the 3′-UTR to regulate neuronal maturation in human cells. miR-124 is a regulator of the transcription silencing REST complex, which represses numerous neuron-specific genes in non-neuronal cells including miR-124 itself [11, 37]. miR-124 also modulates the programmed switch of PTB to nPTB during neuronal differentiation by downregulating PTB expression, which in turn reprograms neuronal-specific AS events ([136] and illustrated in Fig. 2a).

There are also several lines of evidence that PTB dependent downregulation of multiple components of the REST complex occurs in the brain to maintain non-neural cell identity. Multiple PTB binding peaks were observed in the 3′-UTR of key REST complex factors, *CoREST* and *HDAC1*, both of which have been implicated in neurogenesis [241]. In the case of REST complex factor *SCP1*, mutational analysis of binding sites of PTB and miR-124 demonstrated that PTB directly competes with miR-124 on its target site in the 3′-UTR of the *SCP1* gene. miR-124, REST and PTB form an intricate regulatory loop that induces significant AS reprogramming affecting the fate of neuronal lineage [59, 95].

In other instances, PTB has been observed to enhance miRNA targeting by inducing structural changes to the mRNA. *GNPDA1* is upregulated by PTB via its 3′-UTR. PTB binding sites in the 3′-UTR of *GNPDA1* transcripts are immediately downstream of potential targeting sites for several miRNA, including Let-7b, miR-181b and miR-196a. Luciferase reporter assays have demonstrated enhanced GNPDA1 expression in a PTB-dependent manner. The mechanism for PTB-dependent enhancement of miRNA action relies on a stem-loop; this demonstrates a dynamic switch between the single-stranded and double-stranded states. The presence of PTB enhances the single-strandedness of the stem-loop, exposing the region that houses miRNA target sites. PTB can potentially modulate RNA secondary structure that may expose or shield miRNA target sites in the region [241].

## RNA EDITING

RNA editing is widespread in mRNAs of higher eukaryotes and more specifically, editing by Adenosine deaminase acting on RNA (ADAR) RBPs has been found to be most abundant in the nervous system [6, 123]. RNA editing has profound effects on the transcriptome and concordantly the proteome, demonstrating another mechanism through which biological complexity is achieved (reviewed in [65, 161]). Mammals contain three types of ADARs, ADAR1 (primary editor of repetitive sites), ADAR2 (primary editor of non-repetitive coding sites) and the catalytically inactive ADAR3, which predominantly acts as an inhibitor of editing. ADARs catalyze the deamination of adenosine (A) into inosine (I) (known as A-to-I editing) in double-stranded RNA post-transcriptionally and this form of mRNA editing is highly prevalent (reviewed in [16]). Inosine is read as guanosine by the cellular machinery, thereby changing the identity of the encoded protein by altering a codon or mRNA regulatory site (reviewed in [160]).

**Figure 3:**
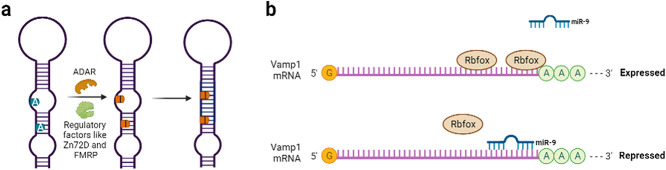
(a) ADAR mediated RNA editing. ADAR mediates the conversion of A-to-I in mRNAs and factors like Zn72D and FMRP regulates this process (adapted from [17, 187, 218]). (b) Translational control of Vamp1 mRNA. Rbfox and miR-9 competitively approach the binding site in the 3′-UTR of Vamp1 mRNA. Binding of Rbfox enhances the mRNA stability and aids translation, whereas binding of miR-9 represses translation of Vamp1 (adapted from [222]).

A-to-I editing is known to alter the critical properties of neuronal proteins and is consequently important for synaptic transmission (reviewed in [206]). The catalytically inactive ADAR3 represses RNA editing by competitively binding to target mRNAs in both human cells and *Caenorhabditis elegans* [163, 179, 229]. ADAR2 in particular is responsible for editing several mammalian pre-mRNAs encoding ion channels involved in regulating neuronal excitability (reviewed in [184]). The dysregulation of ADAR2 is involved in many neurological diseases (reviewed in [196]). ADAR2 contains two functionally redundant nuclear localization signals, which elevate its nuclear localization and expression. ADAR2 subcellular localization has been demonstrated to be exclusively nuclear that may explain the crucial role of editing during neuronal development and maturation [53, 186]. ADAR mediated RNA editing is illustrated in Fig. 3a.

The *Drosophila melanogaster Adar*, a human Adar2 homolog, regulates sleep by altering synaptic plasticity. Data indicates that *Adar* suppresses glutamatergic signaling to suppress sleep. *Adar* knockout specifically upregulates vesicular glutamate transporter and hyperactivates NMDA signaling leading to the sustained release of neurotransmitters. This probably could increase synaptic potentiation associated with sleep-promoting neurons and thus promote sleep in *Adar* mutants. Additionally, *Adar* mutants affect neuronal structure and physiology. The consequences of loss of *Adar* activity also include abnormal circadian motor patterns and male courtship behaviors [22, 106, 137, 183]. *Caenorhabditis elegans* contains a single ADAR enzyme, the neural ADR-2. A novel technique that combines neural cell isolation with RNA-sequencing and editing site detection with software for accurately identifying locations of RNA editing software has identified over 7300 editing sites, with 104 novel edited genes in the nervous system transcriptome of *C. elegans*. A differential expression analysis in neural cells following the loss of *adr-2* has implicated the *clec-41* gene in the regulation of proper chemotaxis. The *clec-41* transcript was shown to be edited in the 3′-UTR region and neural transgenic expression of *clec-41* in *adr-2* deficient *C. elegans* was sufficient to rescue the aberrant chemotaxis, displaying a tissue-specific role of ADR-2 [50]. Such significance of RNA editing necessitates a better understanding of how these processes are regulated.

### Regulation of RNA editing by RBPs

Global changes in RNA editing have been reported in disease and development, necessitating a better understanding of this means of regulation. While ADAR proteins are well-known regulators of the human editomes (reviewed in [161]). ADAR editing only accounts for some of the editing variations that differ across tissues and development [28, 204, 228]. Concordantly, several RBPs have been found to mediate RNA editing providing additional means for diversifying editing mechanisms via *trans*-regulation. RBPs such as RPS14 and SFRS9 modulate ADAR2 activity. They exhibit substrate-specific RNA binding that inhibits editing of the substrates. It may be possible that these factors are involved in direct competitive binding at or near the ADAR2 editing site. They also show direct interaction and colocalization with ADAR2 that could mean they form complexes with ADAR2 at specific editing sites [205].

The RBP zinc-finger protein at 72D (Zn72D) was identified as a broadly influential RNA editing regulator. *Zn72D* knockdown causes a decrease in ADAR protein levels in the *D. melanogaster* brain that leads to mRNA editing and splicing changes accompanied by defects at the neuromuscular junction and hence impaired locomotion in *D. melanogaster* [187, 237]. ADAR and Zn72D colocalize in the nuclei of brain tissue and co-immunoprecipitate together indicating that they physically interact with one another. Many edited transcripts also immunoprecipitated with *D. melanogaster* Zn72D. The ADAR and Zn72D interaction within the nucleus appears to be RNA-dependent, suggesting that they either bind the same RNAs or are part of the same ribonucleoprotein (RNP) complexes. In the *D. melanogaster* brain, Zn72D appears to regulate both splicing and/or editing in different subsets of transcripts [187].

In addition to the findings in *D. melanogaster*, Zn72D mouse homolog, Zfr, has also been implicated in RNA editing in primary cortical neurons. Zn72D, the human homolog of Zfr has been reported to influence pre-mRNA splicing and RNA editing suggesting that this mode of editing regulation is highly conserved and elucidation of mechanistic details of these processes may contribute significantly to therapeutic interventions [79, 90, 187].

### FMRP family of RBPS in RNA editing

The fragile X mental retardation 1 (FMRP) family of RBPs affects RNA editing in multiple organisms [22, 76, 179, 193]. Loss of *FMRP* protein function leads to heritable intellectual disability. While FMRP is a RBP typically associated with translational repression, *D. melanogaster* fragile X homolog (dFMR1) has also been implicated in RNA editing via dADAR. dFMR1 biochemically interacts with dADAR and this interaction seemingly affects the RNA editing of several dADAR targets. These targets happen to have key roles in the modulation of the synaptic architecture of neuromuscular junction and synaptic transmission suggesting that dFMR1 regulation of dADAR activity is essential for the aforementioned functions. dFMR1 and dADAR are both RBPs that may associate in a common complex on shared RNA targets, this is further bolstered by immunoprecipitation experiments that demonstrate association of dFMR1 and dADAR on common RNA substrates. These findings link FMRP to novel neuronal functions via the RNA editing pathway [22].

Hypoediting of RNA in ASD subjects is a common trend across different brain regions. FXR1 is an RBP involved in the regulation of memory and emotions [39, 52]. FXR1 also reduces RNA editing in the brain in a cell type-specific manner and contributes to hypoediting in autism brains [211]. Immunoprecipitation experiments demonstrates that FXR1P binds to the regions containing ADAR1 sites and mutation of predicted FXR1 binding motifs causes higher editing levels at most target sites. These results assign roles of direct inhibitory regulation of editing sites to FXR1P through its interaction with ADAR1 and the RNA. In contrast, FMRP displays editing enhancing roles, demonstrating concomitant modulation of RNA editing by these two RBPs at several editing sites. Mutation or loss of FMRP binding sites in target RNA causes significant reduction in RNA editing indicating that FMRP interacts with RNA and ADAR to mediate editing. A vast number of studies implicate the involvement of FMRP in the pathogenesis of ASD, necessitating the elucidation of its molecular mechanisms [48, 72, 87, 88, 102, 105, 168, 174, 211].

The RNA binding sites of FMRP and FXR1P are significantly enriched around editing sites of transcripts in the frontal cortex of ASD patients, suggesting their role as direct regulators of RNA editing in ASD. Furthermore, FMRP and FXR1P interact with one another suggesting a synergistic regulation of RNA editing [250]. Western blot analysis shows that FMRP expression is absent or reduced in Fragile X syndrome patient brain samples, while *ADAR1* and *ADAR2* expression levels remain similar. Several lines of evidence, such as these, indicate that the dysregulation of RNA editing in Fragile X syndrome and ASD occur through common means involving FMRP regulation of RNA editing. This establishes a correlation between hypoediting and *FMR1* and *FXR1* genes demonstrating a shared RNA editing patterns and molecular deficit between two closely related neurodevelopmental disorders, ASD and Fragile X syndrome [211].

## REGULATION OF RNA STABILITY, DECAY AND TRANSLATION BY RBPS

Post-transcriptional gene expression in the nervous system is tightly controlled by *trans*- acting elements such as RBPs and non-coding RNAs. This regulation affects vital processes such as splicing, translation, RNA transport, RNA stability and decay. Interdependent networks of RBPs may regulate complex pathways involved in various aspects of neuronal development and functioning. Hence, elucidation of the dynamics within RBP networks may shed light on the regulation of key neuronal processes such as neurogenesis, synaptic transmission and synaptic plasticity.

Rbfox1 regulates RNA metabolism of hundreds of genes in neurons [86, 117, 233]. Dysregulation of *RBFOX1* has been linked to intellectual disability, autism, epilepsy and Parkinson’s disease (PD) pathologies that are shared with dysregulation of brain-derived neurotrophic factor (BDNF) signaling (reviewed in [23, 38]). BDNF is secreted during long-term potentiation (LTP) induction and is essential for the signaling leading up to LTP, making it a potent mediator of synaptic plasticity (reviewed in [134, 167]). Concordantly, it has key roles in cognitive functions [12, 167]. Abnormal activity of BDNF receptor Ntrk2 (TrkB) also impairs LTP and reduces synapse numbers that hinder hippocampus-dependent memory formation and consolidation (reviewed in [149]). The *Ntrk2* gene generates different TrkB isoforms including full-length tyrosine kinase receptor (TrkB.FL) and truncated receptors sans the kinase domain (TrkB.T1). Dysregulation of TrkB isoforms expression is associated with neuropsychiatric and neurodegenerative disorders [57, 61, 67, 75].

Rbfox1 specifically regulates TrkB.T1 receptor levels in neurons. Overexpression of Rbfox1 impairs BDNF-dependent LTP and this defect can be rescued by restoring TrkB.T1 levels. Rbfox1 upregulation causes an increase in hippocampal TrkB.T1 isoform expression and as a result, impairs BDNF-dependent LTP, suggesting that Ntrk2 or TrkB is an important Rbfox1 target [78, 86, 117, 216]. Rbfox1 was the first factor shown to regulate Ntrk2 expression at the mRNA level. It directly binds to *Ntrk2* RNA and promotes TrkB.T1 RNA stability, rather than AS. This notion is supported by Rbfox1 overexpression studies that only lead to the up-regulation and stability of TrkB.T1 RNA and has no effect on TrkB.FL; this is further confirmed by RNA-seq analysis displaying changed expression of only TrkB.T1 in a Rbfox1 overexpression background in mouse hippocampus. Another important discovery was that despite the presence of six ‘GCATG’ Rbfox1 binding sites in the 3′-UTR, the influence of RbFox1’s expression on TrkB.T1 mRNA levels appears to be independent of its action on the 3′-UTR region. This suggests that TrkB.T1 mRNA isoform may be regulated by Rbfox1 through different processes [210].

RNA-seq analysis shows that gain or loss of function of Rbfox1 seem to regulate different genetic landscapes indicating that Rbfox1 has broad genetic targets. Upregulation of Rbfox1 leads to an increased expression of TrkB.T1 that impairs BDNF-induced LTP. Surprisingly, reduction in Rbfox1 level does not have an effect TrkB.T1 expression. Most studies have assessed the consequences of *RBFOX1* downregulation; however, upregulation of *RBFOX1* is also tied to specific pathologies. For instance, iPSCs derived neurons from of PD patients have elevated RBFOX1 levels [129]. Interestingly, neurons of PD patients also display increased TrkB.T1 expression, which may indicate impairment of similar regulatory mechanism on TrkB.T1 in PD patients [73]. These studies indicate that upregulation or downregulation of specific RBPs could have entirely different genetic outcomes.

Cytoplasmic Rbfox1 promotes mRNA stability and/or translation by binding to a conserved motif in the 3′-UTRs of target transcripts and is highly enriched in the brain [30, 117]. In the hippocampus, vSNARE protein Vamp1 is an important cytoplasmic Rbfox1 target. Vamp1 is expressed specifically in inhibitory neurons, and loss of *Vamp1* and *Rbfox1* causes decreased inhibitory synaptic transmission and excitatory/inhibitory imbalance. In *Rbfox1 Nes-cKO* mice, loss of binding by RBFOX1 to Vamp1 3′-UTR strongly downregulated Vamp1 expression. Cytoplasmic Rbfox1 induces Vamp1 expression in part by blocking *microRNA-9*. RBFOX1 binding to Vamp1 3′-UTR increased mRNA levels possibly by promoting mRNA stability and/or translation along with antagonizing *miR-9* action. Rbfox1 and *miR-9* regulatory networks may modulate the expression of Vamp1 in an opposing manner to control inhibitory synaptic transmission during learning and memory. Cytoplasmic Rbfox1 largely affects a different set of transcripts from those regulated by nuclear rbfox1 highlighting additional post-transcriptional regulatory programs performed by the Rbfox family of RBPs aside from AS [222]. The translational control of Vamp1 mRNA is illustrated in Fig. 3b.

### Memory consolidation via external sensory cues

Different patterns of stimulation alter LTP, long-term depression or homeostatic synaptic scaling within synaptic connections between neuronal cells. These processes establish memory formation ability as a function of experience (reviewed in [202, 203]). As an example, *C. elegans* sensory neurons are responsible for changes in animal behavior and this requires spatiotemporal control of regulated neuronal protein synthesis, which is said to adjust the synaptic connection strength as a function of experience. *Caenorhabditis elegans* responds to over 60 different attractant volatile chemical compounds via its AWA and AWC olfactory sensory neurons that mediate attraction and chemotaxis toward the odor [13]. Sensitivity to an odor depends on prior exposure time, brief exposure causes short-term adaptation and prolonged/repeated exposure causes long-term adaptation where the animals will ignore the odor [13, 36, 115].

Short- and long-term adaptation in *C. elegans* is facilitated by AWC sensory neurons that require the cGMP-dependent protein kinase EGL-4 and translational control of *egl-4* mRNA by pumilio/Fem-3 binding factor (PUF) family of regulatory RBPs, essential for suitable behavioral response [115]. PUF proteins are typically categorized as translational repressors with few instances of promoting translational activation. For instance, *D. melanogaster* Pumilio mediated translational repression affects neuronal membrane excitability, synaptic development, dendritic branching and plasticity [145, 146, 243]. *Drosophila melanogaster* pumilio is also required for olfactory associative learning [60].

The 3′-UTR of *egl-4* mRNA contains a highly conserved nanos response element that binds to the FBF-1 PUF protein. This specific binding event promotes adaptation to the odors, butanone and isoamyl alcohol and causes an increase in EGL-4 expression. FBF-1 directly activates translation of its target via its conserved 3′-UTR binding site in response to stimulation in adult functioning sensory neurons. The 3′-UTR of *egl-4* also contains miRNA seed sequence 3′ of nanos response element, which enhances basal EGL-4 expression. In addition to the possible role of miRNAs in positively affecting EGL-4 translation in collaboration with FBF-1, FBF-1 may also help localize EGL-4 translation near the AWC sensory cilia [83]. Nervous system-wide presynaptic transcriptome of *C. elegans* has previously revealed that mRNAs for pumilio RBPs are abundant in synaptic regions. Presynaptic PUMILIOs have also been found to regulate associative memory [5]. *Caenorhabditis elegans* have 11 PUF proteins that interact with PUF protein partners such as NOS-1 and cytoplasmic poly(A) polymerase GLD-3, which may promote adaptation to other AWC-sensed odors via different targets [63, 225, 234].

Humans have two pumilio proteins, PUM1 and PUM2 [197]. These two proteins regulate a highly overlapping but non-identical set of mRNA targets. Mammalian pumilio proteins have been implicated in proper neuronal activity, ERK signaling, germ cell development and stress response, making them important factors in regulating neuronal functions [83, 118, 152, 209, 219].

### Role of long non-coding RNAs in regulating neuronal transcripts and memory

Recent advances in RNA seq analysis have demonstrated beyond doubt that the neuronal transcript pool is enriched with non-coding RNAs (reviewed in [185]). ncRNAs could be clustered into two groups based on their length. Small non-coding RNAs are less than 200 bases long and comprises of miRNA, nucleolar RNA and Piwi RNA. On the other hand, a significant proportion of ncRNA is longer than 200 bases and comprises long non-coding RNA (lncRNA) and circular RNA (reviewed in [125]).

**Figure 4:**
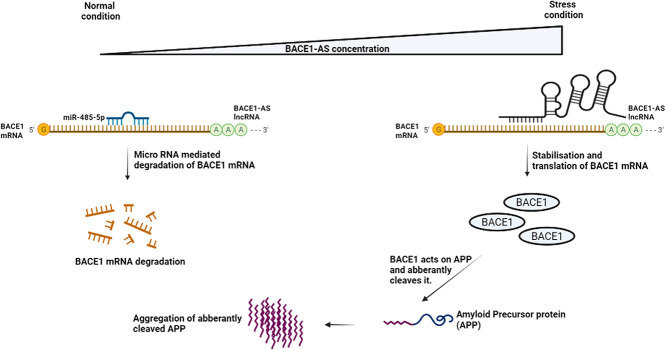
Role of BACE1-AS lncRNA in driving APP aggregation. BACE1 is an enzyme that cleaves APP aberrantly and drives aggregation. Under normal conditions, BACE1 expression is lower due to miRNA (miR-485-5p) mediated degradation of *bace1* mRNA. However, during stress the expression of BACE1-AS is upregulated. BACE1-AS competes for the same site as miR-485-5p and successful binding of the lncRNA confers stability to *bace1* mRNA and promotes translation. This image is based on work presented in [68, 69].

The lncRNA is usually processed by RNA polymerase 2 and contains 5′ capping and 3′ poly A tailing similar to that of coding transcripts (reviewed in [125, 199]). Most lncRNAs are localized in the nucleus and play a significant role is chromatin remodeling ([148] and reviewed in [89]). However, some lncRNAs are cytoplasmic and play a crucial role in transcriptional control (reviewed in [247]). One of the most well-studied lncRNA involved in PTR is BACE1-AS. AS refers to anti-sense, i.e. BACE1-AS is transcribed from the anti-sense strand of the gene encoding BACE1. BACE1 codes for an enzyme, beta secretase, which catalyzes the aberrant cleavage of amyloid precursor protein (APP), leading to APP aggregation, a hallmark of Alzheimer’s disease. Typically, lower levels of BACE1 are preferred and maintained and this regulation is achieved through a miRNA, miR-485-5p, which induces translational repression of the BACE1 transcript. In contrast, the lncRNA BACE1-AS competes for the same sites as miR-485-5p and successful binding of BACE1-AS to BASE-1 mRNA enhances stability and promotes translation [69]. Higher levels of BACE1 increase the concentration of aberrantly cleaved APP that undergoes aggregation and affects neuronal signal transduction. Under normal conditions the concentrations of BACE1-AS and miR-485-5p are fine-tuned, whereas under stress or Alzheimer’s disease, BACE1-AS is overexpressed and as a result the translational levels of BASE1 are upregulated [68]. This function of BASE1-AS is illustrated in Fig. 4.

## LOCALIZATION AND TRANSPORT OF RNA

Pioneering work published in 1988 firmly established the polarity of neurons. Their highly polarized nature is typically exhibited via a single axon and several dendrites that can extend structurally and functionally distinct processes far from the cell body (soma). The dendrites receive signals at the synapse and relay it to the soma that in turn propagates it along the axon to intended presynaptic sites. Thus, the polarized morphology of neurons is important for the directional flow of information in the nervous system [58].

Neurons rely on protein synthesis as they are essential sensors and effectors to communicate with different cell types. These proteins establish synaptic connections, changes in plasticity, memory and information storage. Neurons can extend hundreds of centimeters in length and respond to stimuli in remote regions within milliseconds. This is possible due to the rapid proteome modifications in axons and dendrites. These almost instantaneous metabolic changes rely on several mechanisms that facilitate local protein synthesis. These mechanisms dictate messenger RNA transport from the soma to distal ends of the neuron. Translationally silent mRNAs are targeted to synapses and subsequently lead to local synthesis of proteins. RNA transport is essential for local protein synthesis in neurons that allow for access to necessary proteins at distant sites (reviewed in [74]).

The elaborate architecture of neurons requires specialized machinery that is constantly regulated for localization and transport. Despite the significance of mRNA movement in neurons, we do not yet fully understand how these events are regulated. While we do not have a well-established model, various regulatory motifs in the mRNA and a plethora of *trans*-elements that bind to them work together to achieve localization. Anomalous RNA transport can lead to dire consequences with respect to several neurodegenerative diseases. The aftermath of transcription sees RNAs subjected to several modifications that make them attractive targets for *trans*-elements ([70, 133] and reviewed in [74]).

Additionally, the UTRs of mRNAs can potentially alter their targeting, stability and translational modulation. In microdissected rat brain slices, 3′-UTRs of transcripts localized at the axons, dendrites and synapses are significantly longer with longer half-lives than somata-enriched transcripts. Long 3′-UTRs are a hotbed of *cis*-elements that interact with *trans*-regulatory factors [215]. While the average length of the 5′-UTR of mRNAs is significantly shorter than the 3′-UTR in humans, mutations in 5′-UTR are known to impair localization. The 5′-UTRs also harbor RBP binding sites and other regulatory elements including IRES, uAUGs and uORFs that dictate translational efficiency [121, 147].

In previously published data on genes expressed in synaptic regions, relative to somatic regions in the *C. elegans* adult central nervous system, revealed presynaptic enrichment of mRNA associated with 3′-UTR binding (RBPs) and translation regulator activity [5]. This may provide an additional layer of translation control in the neurons that is stimulus-specific. Of the several RBP transcripts that were enriched in the synapse, *pufs* (*puf-3, puf-5, puf-7, puf-8, puf-11*) were the most numerous. Synaptically localized *pufs* were also shown to be necessary for normal positive olfactory associative memory formation. These *puf* RBPs are orthologs of mammalian PUM1/2 both of which are known to interact with FMRP in a collaborative, RNA-dependent manner with possible roles in synaptogenesis [170, 249]. While PUM2 has a key role in mRNA localization and translation where it restricts specific transcripts to the cell body in developing neurons, the enrichment in neurons of adult animals allude to their roles in plasticity and behavior in the axonal and presynaptic regions [141].

The importance of *cis*-regulatory elements in the UTRs is demonstrated in the transport of neuritin mRNA (Nrn1). Nrn1 promotes synapse formation and maintenance [81, 154, 158]. Its mRNA localizes to axons in dorsal root ganglion (DRG) and hippocampal neurons, this localization changes following nerve injury [207, 236]. Nrn1 transcript localization increases in axons and decreases in the soma when subjected to axotomy. Moreover, Nrn1 3′-UTR drives its axonal localization in the hippocampal neurons (CNS); it is the 5′-UTR that is essential for the same in in DRG neurons (PNS), the latter of which promotes neurite growth post injury. These distinct events may be explained by different RBPs binding to the Nrn1 5′-UTR versus Nrn1 3′-UTR. For instance, survival of motor neuron (SMN) and HuD immunoprecipitated together while Nrn1 mRNA colocalizes with SMN in hippocampal axons making HuD a prime Nrn1 3′-UTR regulatory RBP candidate. Similarly, different RBPs may also associate with Nrn1 5′-UTR for axonal localization in the DRG neurons [147].

*Trans*-elements such as ZBP1/IGF2BP1 are a great example of RBPs’ role in cellular transport. It is highly expressed in the embryo and is responsible for the development of the nervous system. Defects in ZBP1 gene expression in developing neurons are associated with growth cone guidance, axonal remodeling, dendritic morphology defects, as well as smaller cerebral cortices [66, 122, 144, 162, 188, 231]. This RBP is well known for its regulation of local translation of *β-actin* mRNA. ZBP1 associates with *β-actin* mRNA in perinuclear space through its two C-terminal KH domains that bind to *β-actin* 3′-UTR ‘zipcode’ 54-nucleotide RNA sequence. ZBP1 KH domains, KH3 and KH4 form a pseudo-dimer with the two RNA-binding grooves on opposite sides [71, 171]. This binding event is accompanied by the RNA molecule looping around the protein leading to translationally repressed transcripts that remain so until they reach their destination at the cell periphery. At this point, the protein kinase Src phosphorylates ZBP1 to sever the ZBP1-RNA complex and promote translation [100, 238]. Thus, ZBP1 prevents premature translation of the transcript demonstrating the spatiotemporal aspect of mRNA localization and translation [159].

**Figure 5:**
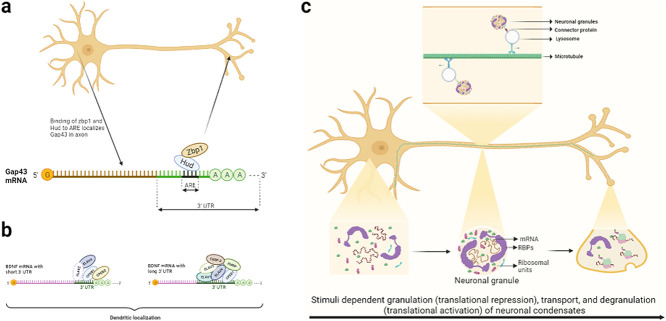
(a) Localization of GAP-43 mRNA in axons. Binding of Hud and Zbp1 to the ARE element in the 3′-UTR of GAP-43 transports the mRNA to axons (adapted from [246]). (b) Localization of BDNF mRNA in dendrites. Based on the length of BDNF mRNA 3′-UTR, the factors required for dendritic localization vary. CPEB-1, CPEB-2, ELAV-2 and ELAV-4 are crucial for the dendritic localization of BDNF mRNA with short 3′-UTR, whereas CPEB1, ELAV1, ELAV3, ELAV4, FMRP and FXRP2 are required for the localization of BDNF mRNA with long 3′-UTR (adapted from data presented in [220]). (c) Formation of neuronal granules. Neuronal granules, which are transported from the soma to the nerve ending, are formed by non-covalent interaction between mRNAs and RBPs (adapted from [74]).

ZBP1 is also required for axonal localization of GAP-43 mRNA, important for axon growth and regeneration [55, 56]. GAP-43 mRNA coimmunoprecipitates with both ZBP1 and the ELAV-like RBP HuD that binds to an AU-rich regulatory element (ARE) in the 3′-UTR of GAP-43 mRNA and promotes local stabilization [19, 150]. While GAP-43 mRNA does not have a distinct ZBP1 binding zipcode, HuD binding to GAP-43 ARE is necessary and sufficient for GAP-43 mRNA axonal localization. ZBP1 and HuD RBPs post-transcriptionally regulate GAP-43 mRNA by forming a complex via the ARE element [246]. This is illustrated in Fig. 5a.

Complex mechanisms of localization and transport have been unearthed previously. For instance, dendritic trafficking of BDNF transcripts displays both activity dependence and transcript selectivity. Transcripts of neurotrophin BDNF and its receptor TrkB are targeted to the dendrites in cultured hippocampal neurons. BDNF is involved in regulating synaptic plasticity by enhancing synaptic transmission and is involved in hippocampal LTP (reviewed in [25, 208]). BDNF is also a well-known regulator of dendritic patterning and morphology. *BDNF* transcripts exist in two types of isoforms, either with long or short 3′-UTRs, as a result of its gene being processed at two different polyadenylation sites. This affects their localization to different subcellular compartments and the 3′-UTRs also carry *cis*-dendritic targeting signals. Transcripts with short 3′-UTRs are restricted to the soma under resting conditions, in the absence of specific neuronal cues, while those with long 3′-UTRs are targeted to both the soma and dendrites [3, 116, 220]. These differential targeting mechanisms rely on separate sets of RBPs functioning in an activity-dependent manner [1, 9, 34, 35, 165, 220, 235].

Transcripts with short 3′-UTRs are transported to the dendrites, induced by neuronal stimuli and this dendritic localization is mediated by neurotrophin NT3 and requires the RBP, cytoplasmic polyadenylation element binding protein-1 (CPEB-1) [9, 165]. The short 3′-UTR contains cytoplasmic polyadenylation element (CPE)-like *cis*-acting motifs to which CPEB-1 binds directly. As CPE-like sequences were shown to be necessary for BDNF mRNA dendritic targeting to hippocampal neurons, CPEB-1 may have a key role in BDNF mRNA targeting. In addition to CPE elements, short 3′-UTRs also contain conserved ELAV RBP binding sites, disruption of which hinders short BDNF 3′-UTR dendritic targeting. BDNF mRNA localization to dendrites seems to rely on its association with CPEB-1, CPEB-2, ELAV-2 and ELAV-4 RBPs. In contrast, long 3′-UTR targeting is more complex and relies on ELAV-1,3,4, FMRP and FXRP2 mediated BDNF-dependent release of soma-retention signals. Long 3′-UTRs also contain a highly conserved CPEB-1 binding site. Thus, BDNF mRNA dendritic targeting mechanisms are induced by different stimuli and associate with distinct sets of RBPs [165]. This is illustrated in Fig. 5b.

The coding sequence also plays pivotal roles in these mechanisms. Translin, a single-stranded DNA/RNA binding protein binds directly to the coding sequence of BDNF mRNA and this binding event is associated with constitutively active dendritic targeting of transcripts with both short and long 3′-UTRs. Translin-dependent transport of transcripts restricted to the soma can be overridden by 5′-UTR *cis*-elements [1, 9, 10, 34, 35, 165, 172].

Similarly, the 3′-UTR of *CaMKIIα* transcripts are present in short or long forms and have two CPE elements that promote mRNA transport and translational activation in dendrites. The coding sequence also contains translin-binding sites necessary for dendritic localization [98, 191]. These studies hint at a common mRNA targeting mechanism shared by different mRNAs with common functions, which utilize *cis*-elements that associate with distinct set of RBPs.

## NEURONAL GRANULES

Neuronal granules are membrane delimited phase-separated condensates, which aid the transport of mRNA to the far ends of neurons including dendrites, axons and synapses from the cell body, to aid local translation of signaling molecules. These condensates are formed by non-covalent interactions between mRNAs and RBPs. The neuronal mRNAs that are to be transported to the distant end of neurons consist of comparatively longer UTRs. These regions act as hotspots for the binding of RBPs and other *trans*-factors. Apart from binding to mRNA, some of the RBPs consist of intrinsically disordered regions [214]. Studies have shown that these low complexity regions undergo assembly through pi-pi and other non-covalent interaction leading to phase separation ([217] and reviewed in [74]). Neuronal granules are illustrated in Fig. 5c.

Neuronal granules delimit themselves from the rest of the cytosol, making the mRNA inside the granule inaccessible to the cytoplasmic translational machinery. Interestingly, RBPs like Smaug1, ZBP1 themselves repress the translation of mRNA to which they are bound ([7], Anderson). More recent studies have suggested that the nuclear granules are actively transported bidirectionally. The anterograde and retrograde movement is achieved by binding of the nuclear granules to the membrane bound organelles, leading to cotransport with the organelle. Anterograde movement helps local transport of specific mRNAs involved in presynaptic activity, whereas retrograde movement is crucial for maintaining homeostasis, degradation of aging proteins and organelles in the synapse, recycling and signaling during injury (reviewed in [135]). The attachment of the nuclear granules to the organelles is achieved through specific RBPs like SMN, She2p and Annexin. Specifically, Annexin A11 has a C-terminal domain that tether to membrane lipids, PI(3,5)P2-present in lysosomes-and an N-terminal comprising low complexity regions that aid phase separation (reviewed in [178]). Mutations in annexin A11 in amyotrophic lateral sclerosis have been shown to compromise neuronal granules’ transport by inhibiting the binding of annexin to the lysosome [126].

The neural UTRs are considerably longer than in other cells and provide several sequence motifs for RBPs and miRNA binding. These features along with other RNA transport promoting factors contribute to RNA delivery to distal neuronal destinations. RNAs are mostly found in complexes with RBPs (RNP complexes) and of the nearly 2000 known RBPs in the human genome, some are exclusively or predominantly expressed in neurons with neuron-specific roles (ELAVL, RBFOX, FUS and FMRP) (reviewed in [41, 49]). RNP complexes along with ribosomal components form neuronal transport granules that package mRNA for transport. They exhibit constitutive bidirectional movement along axons and dendrites and selectively release mRNA for translational initiation by polysomes following neuronal depolarization [110, 113]. Transport granules are unique in that they are liquid–liquid phase separated from the rest of the liquid cytosol, unlike other conventional soluble complexes due to the multivalent and dynamic interactions between RNA and protein components with RNA binding domains such as RRM or KH domains and intrinsically disordered regions with low-complexity sequences [27, 130]. These biomolecular condensates form dynamic membrane-less organelles that enclose RNA and protein components and restrict cytosolic accessibility. When this mechanism is impaired, it is associated with several neurodegenerative diseases (reviewed in [29, 74]).

### Regulation of neuronal granule transport

Activity-dependent reversible activation of local translation is important for synaptic strength, plasticity and long-term memory formation (reviewed in [109]. Neuronal stimuli at the synapse quickly induce translation of silenced mRNAs in neuronal transport granules (reviewed in [203]). Activity-dependent translation is thought to require reversible granule formation. For instance, *β-actin* mRNA is packaged into neuronal granules that assemble to remain translationally repressed and disassemble for localized translation due to neuronal stimuli [62].

RBP FMRP is enriched in transport granules and phase separates with RNA into liquid droplets, *in vitro*, via its C-terminal low-complexity disordered region (LCDR) [217]. FMRP has been demonstrated to form membraneless foci in cells and liquid droplets with RNA *in vitro* and the C-terminal FMRP-LCDR is necessary and sufficient for these events *in vitro*. FMRP typically behaves as a translational repressor in a phosphorylation-dependent manner via ribosomal stalling [156, 157]. Additionally, methylation of FMRP RGG motifs may impede ribosomal stalling and decrease its binding affinity toward G-quadruplex-containing RNAs [47, 54, 180]. While numerous studies have elucidated the mechanisms of translational control by FMRP, these mechanisms have not been clearly defined in the context of neuronal transport granules.

The abundance of FMRP in neuronal granules plays a vital role in activity-dependent mRNA translation through post-translational modifications such as phosphorylation and methylation, which lead to opposing effects on translational regulation. FMRP *in vitro* translation inhibition and phase separation was also observed to be FMRP concentration-dependent. This is mediated by post-translational modification where FMRP phosphorylation enhances phase separation and translation inhibition while methylation opposes them. Furthermore, other translational repressors that function alongside FMRP, including neuronal eukaryotic initiation factor 4E binding protein (4E-BP) and miRNA 125b, were sequestered into FMRP-RNA liquid droplets, which may strengthen their inhibitory activity [64, 153, 155, 214].

### Regulation of local translation of silenced MRNAs

Reversible granule formation allows for repeated local translation of the same mRNA transcript that transitions from a repressed state within granules into an active state highlighting the significance of synaptic regulation of phase separation [113]. RBPs and miRNA *trans*-acting elements are ideal for selective and reversible inhibition of mRNA translation at synapses in response to receptor signaling (reviewed in [31, 112]). In fact, RBPs and miRNAs may cooperate to achieve rapid translational regulation [64]. For instance, FMRP associates with mammalian eIF2C2 (AGO2) and miRNAs. FMRP phosphorylation leads to AGO2-miR125a inhibitory complex formation on postsynaptic density protein (PSD-95) mRNA 3′-UTR. In contrast, gp1 mGluR stimulation causes dephosphorylation of FMRP and dissociation of AGO2 from the mRNA followed by translational activation [153].

FMRP is thought to translationally suppress specific mRNAs, including MAP1b, CaMKIIα and Arc (reviewed in [18]). FMRP is linked to the miRNA pathway as it interacts with proteins in the RNA interference silencing complex and with miRNAs [32, 103, 166, 175]. The 3′-UTR of the NMDA receptor subunit NR2A has conserved miR-125b target sequence, marking it for suppression. Additionally, loss of FMRP or AGO1 function leads to upregulation of NR2A 3′-UTR reporter. This suggests that it is regulated by FMRP and miR-125b through its 3′-UTR. NMDA receptor subunit ratio is an important factor in its receptor signaling with even subtle changes in NR2A expression mediated by RBPs and miRNAs influencing [15, 64].

The 4E-BPs bind eIF4E and downregulate the translation of many mRNAs [182]. Cytoplasmic FMRP interacting protein CYFIP1, a 4E-BP binds the cap-binding factor eIF4E and CYFIP1 is also an FMRP-interacting factor [111, 155, 189]. CYFIP1 forms a complex with the FMRP and represses activity-dependent translation through CYFIP1, a new 4E-BPh specific FMRP-target mRNAs such as MAP1B, αCaMKII and APP ([94, 232] and reviewed in [8]). The eIF4E-CYFIP1-FMRP complex is found at the synapses and synaptic activity was demonstrated to release CYFIP1 from eIF4E and 5′ end of specific mRNAs, curbing translational repression [155].

## CONCLUSION

The proper function of the nervous system depends on precise spatiotemporal control of gene expression. Complex post-transcriptional mechanisms play key roles in the regulation of gene expression to ensure proper neuronal development and synaptic plasticity (reviewed in [49, 151]). PTR is essential for the processes involving RNA metabolism including AS, RNA editing, mRNA stability, mRNA localization and translation (reviewed in [40, 120]). Elucidation of PTR of mRNA targets mediated by *trans*-acting factors is a key step toward understanding their significance in neuronal function, homeostasis and disease.

## FUNDING

This work was supported by the Institute of Eminence postdoctoral fellowship grant from Indian Institute of Science, Bangalore (IE/REAC-21-0119.10 to V.D.B.); the Indian Institute of Science, Bangalore intramural funds; the IA grant (IA/S/19/2/504649 to K.B.); the DBT grants (BT/PR24038/BRB/10/1693/2018 and BT/HRD-NBA-NWB/38/2019-20 to K.B.); and the Ministry of Education (MoE/STARS-1/454 to K.B., MoE/STARS-1/454 to J.J.).

## CONFLICT OF INTEREST

None declared.[Supplementary-material sup1]

## Supplementary Material

suppl_data_kvac011

## References

[1] Aliaga EE, Mendoza I, Tapia-Arancibia L. Distinct subcellular localization of BDNF transcripts in cultured hypothalamic neurons and modification by neuronal activation. J Neural Transm (Vienna). 2009;116:23–32 19082527 10.1007/s00702-008-0159-8

[2] Amir-Ahmady B, Boutz PL, Markovtsov V et al. Exon repression by polypyrimidine tract binding protein. RNA. 2005;11:699–716 15840818 10.1261/rna.2250405 PMC1370756

[3] An JJ, Gharami K, Liao GY et al. Distinct role of long 3′ UTR BDNF mRNA in spine morphology and synaptic plasticity in hippocampal neurons. Cell. 2008;134:175–87 18614020 10.1016/j.cell.2008.05.045 PMC2527207

[4] Andreadis A, Gallego ME, Nadal-Ginard B. Generation of protein isoform diversity by alternative splicing: mechanistic and biological implications. Annu Rev Cell Biol. 1987;3:207–42 2891362 10.1146/annurev.cb.03.110187.001231

[5] Arey RN, Kaletsky R, Murphy CT. Nervous system-wide profiling of presynaptic mRNAs reveals regulators of associative memory. Sci Rep. 2019;9:20314 31889133 10.1038/s41598-019-56908-8 PMC6937282

[6] Athanasiadis A, Rich A, Maas S. Widespread A-to-I RNA editing of Alu-containing mRNAs in the human transcriptome. PLoS Biol. 2004;2:e391 15534692 10.1371/journal.pbio.0020391 PMC526178

[7] Baez MV, Luchelli L, Maschi D et al. Smaug1 mRNA-silencing foci respond to NMDA and modulate synapse formation. J Cell Biol. 2011;195:1141–57 22201125 10.1083/jcb.201108159 PMC3246892

[8] Bagni C, Greenough WT. From mRNP trafficking to spine dysmorphogenesis: the roots of fragile X syndrome. Nat Rev Neurosci. 2005;6:376–87 15861180 10.1038/nrn1667

[9] Baj G, Del Turco D, Schlaudraff J et al. Regulation of the spatial code for BDNF mRNA isoforms in the rat hippocampus following pilocarpine-treatment: a systematic analysis using laser microdissection and quantitative real-time PCR. Hippocampus. 2013;23:413–23 23436435 10.1002/hipo.22100

[10] Baj G, Leone E, Chao MV et al. Spatial segregation of BDNF transcripts enables BDNF to differentially shape distinct dendritic compartments. Proc Natl Acad Sci USA. 2011;108:16813–8 21933955 10.1073/pnas.1014168108 PMC3189043

[11] Ballas N, Grunseich C, Lu DD et al. REST and its corepressors mediate plasticity of neuronal gene chromatin throughout neurogenesis. Cell. 2005;121:645–57 15907476 10.1016/j.cell.2005.03.013

[12] Bambah-Mukku D, Travaglia A, Chen DY et al. A positive autoregulatory BDNF feedback loop via C/EBPbeta mediates hippocampal memory consolidation. J Neurosci. 2014;34:12547–59 25209292 10.1523/JNEUROSCI.0324-14.2014 PMC4160783

[13] Bargmann CI, Hartwieg E, Horvitz HR. Odorant-selective genes and neurons mediate olfaction in *C. elegans*. Cell. 1993;74:515–27 8348618 10.1016/0092-8674(93)80053-h

[14] Barnby G, Abbott A, Sykes N et al. Candidate-gene screening and association analysis at the autism-susceptibility locus on chromosome 16p: evidence of association at GRIN2A and ABAT. Am J Hum Genet. 2005;76:950–66 15830322 10.1086/430454 PMC1196454

[15] Barria A, Malinow R. NMDA receptor subunit composition controls synaptic plasticity by regulating binding to CaMKII. Neuron. 2005;48:289–301 16242409 10.1016/j.neuron.2005.08.034

[16] Bass BL. RNA editing by adenosine deaminases that act on RNA. Annu Rev Biochem. 2002;71:817–46 12045112 10.1146/annurev.biochem.71.110601.135501 PMC1823043

[17] Bassell GJ. Fragile balance: RNA editing tunes the synapse. Nat Neurosci. 2011;14:1492–4 22119945 10.1038/nn.2982

[18] Bassell GJ, Warren ST. Fragile X syndrome: loss of local mRNA regulation alters synaptic development and function. Neuron. 2008;60:201–14 18957214 10.1016/j.neuron.2008.10.004 PMC3691995

[19] Beckel-Mitchener AC, Miera A, Keller R et al. Poly(A) tail length-dependent stabilization of GAP-43 mRNA by the RNA-binding protein HuD. J Biol Chem. 2002;277:27996–8002 12034726 10.1074/jbc.M201982200

[20] Begg BE, Jens M, Wang PY et al. Concentration-dependent splicing is enabled by Rbfox motifs of intermediate affinity. Nat Struct Mol Biol. 2020;27:901–12 32807990 10.1038/s41594-020-0475-8 PMC7554199

[21] Bhalla K, Phillips HA, Crawford J et al. The *de novo* chromosome 16 translocations of two patients with abnormal phenotypes (mental retardation and epilepsy) disrupt the A2BP1 gene. J Hum Genet. 2004;49:308–11 15148587 10.1007/s10038-004-0145-4

[22] Bhogal B, Jepson JE, Savva YA et al. Modulation of dADAR-dependent RNA editing by the *Drosophila* fragile X mental retardation protein. Nat Neurosci. 2011;14:1517–24 22037499 10.1038/nn.2950 PMC3225737

[23] Bill BR, Lowe JK, Dybuncio CT et al. Orchestration of neurodevelopmental programs by RBFOX1: implications for autism spectrum disorder. Int Rev Neurobiol. 2013;113:251–67 24290388 10.1016/B978-0-12-418700-9.00008-3 PMC4318517

[24] Black DL. Mechanisms of alternative pre-messenger RNA splicing. Annu Rev Biochem. 2003;72:291–336 12626338 10.1146/annurev.biochem.72.121801.161720

[25] Bonhoeffer T. Neurotrophins and activity-dependent development of the neocortex. Curr Opin Neurobiol. 1996;6:119–26 8794047 10.1016/s0959-4388(96)80017-1

[26] Boutz PL, Stoilov P, Li Q et al. A post-transcriptional regulatory switch in polypyrimidine tract-binding proteins reprograms alternative splicing in developing neurons. Genes Dev. 2007;21:1636–52 17606642 10.1101/gad.1558107 PMC1899473

[27] Brangwynne CP, Eckmann CR, Courson DS et al. Germline P granules are liquid droplets that localize by controlled dissolution/condensation. Science. 2009;324:1729–32 19460965 10.1126/science.1172046

[28] Brummer A, Yang Y, Chan TW et al. Structure-mediated modulation of mRNA abundance by A-to-I editing. Nat Commun. 2017;8:1255 29093448 10.1038/s41467-017-01459-7 PMC5665907

[29] Calabretta S, Richard S. Emerging roles of disordered sequences in RNA-binding proteins. Trends Biochem Sci. 2015;40:662–72 26481498 10.1016/j.tibs.2015.08.012

[30] Carreira-Rosario A, Bhargava V, Hillebrand J et al. Repression of pumilio protein expression by Rbfox1 promotes germ cell differentiation. Dev Cell. 2016;36:562–71 26954550 10.1016/j.devcel.2016.02.010 PMC4785839

[31] Chang S, Wen S, Chen D et al. Small regulatory RNAs in neurodevelopmental disorders. Hum Mol Genet. 2009;18:R18–26 19297398 10.1093/hmg/ddp072 PMC2657940

[32] Cheever A, Ceman S. Phosphorylation of FMRP inhibits association with dicer. RNA. 2009;15:362–6 19155329 10.1261/rna.1500809 PMC2657015

[33] Chen Y, Zubovic L, Yang F et al. Rbfox proteins regulate microRNA biogenesis by sequence-specific binding to their precursors and target downstream dicer. Nucleic Acids Res. 2016;44:4381–95 27001519 10.1093/nar/gkw177 PMC4872098

[34] Chiaruttini C, Sonego M, Baj G et al. BDNF mRNA splice variants display activity-dependent targeting to distinct hippocampal laminae. Mol Cell Neurosci. 2008;37:11–9 17919921 10.1016/j.mcn.2007.08.011

[35] Chiaruttini C, Vicario A, Li Z et al. Dendritic trafficking of BDNF mRNA is mediated by translin and blocked by the G196A (Val66Met) mutation. Proc Natl Acad Sci USA. 2009;106:16481–6 19805324 10.1073/pnas.0902833106 PMC2752540

[36] Colbert HA, Bargmann CI. Odorant-specific adaptation pathways generate olfactory plasticity in *C. elegans*. Neuron. 1995;14:803–12 7718242 10.1016/0896-6273(95)90224-4

[37] Conaco C, Otto S, Han JJ et al. Reciprocal actions of REST and a microRNA promote neuronal identity. Proc Natl Acad Sci USA. 2006;103:2422–7 16461918 10.1073/pnas.0511041103 PMC1413753

[38] Conboy JG. Developmental regulation of RNA processing by Rbfox proteins. RNA. 2017;8:e1398 10.1002/wrna.1398 PMC5315656 27748060

[39] Cook D, Nuro E, Jones EV et al. FXR1P limits long-term memory, long-lasting synaptic potentiation, and *de novo* GluA2 translation. Cell Rep. 2014;9:1402–16 25456134 10.1016/j.celrep.2014.10.028 PMC4254574

[40] Corbett AH. Post-transcriptional regulation of gene expression and human disease. Curr Opin Cell Biol. 2018;52:96–104 29518673 10.1016/j.ceb.2018.02.011 PMC5988930

[41] Corley M, Burns MC, Yeo GW. How RNA-binding proteins interact with RNA: molecules and mechanisms. Mol Cell. 2020;78:9–29 32243832 10.1016/j.molcel.2020.03.011 PMC7202378

[42] Costessi L, Devescovi G, Baralle FE et al. Brain-specific promoter and polyadenylation sites of the beta-adducin pre-mRNA generate an unusually long 3′-UTR. Nucleic Acids Res. 2006;34:243–53 16414955 10.1093/nar/gkj425 PMC1326019

[43] Coutinho-Mansfield GC, Xue Y, Zhang Y et al. PTB/nPTB switch: a post-transcriptional mechanism for programming neuronal differentiation. Genes Dev. 2007;21:1573–7 17606635 10.1101/gad.1575607

[44] Dai J, Aoto J, Sudhof TC. Alternative splicing of presynaptic neurexins differentially controls postsynaptic NMDA and AMPA receptor responses. Neuron. 2019;102:993–1008 31005376 10.1016/j.neuron.2019.03.032 PMC6554035

[45] Dalpe G, Leclerc N, Vallee A et al. Dystonin is essential for maintaining neuronal cytoskeleton organization. Mol Cell Neurosci. 1998;10:243–57 10.1006/mcne.1997.0660 9618216

[46] Damianov A, Ying Y, Lin CH et al. Rbfox proteins regulate splicing as part of a large multiprotein complex LASR. Cell. 2016;165:606–19 27104978 10.1016/j.cell.2016.03.040 PMC4841943

[47] Darnell JC, Jensen KB, Jin P et al. Fragile X mental retardation protein targets G quartet mRNAs important for neuronal function. Cell. 2001;107:489–99 11719189 10.1016/s0092-8674(01)00566-9

[48] Darnell JC, Van Driesche SJ, Zhang C et al. FMRP stalls ribosomal translocation on mRNAs linked to synaptic function and autism. Cell. 2011;146:247–61 21784246 10.1016/j.cell.2011.06.013 PMC3232425

[49] Darnell RB. RNA protein interaction in neurons. Annu Rev Neurosci. 2013;36:243–70 23701460 10.1146/annurev-neuro-062912-114322 PMC3889695

[50] Deffit SN, Yee BA, Manning AC et al. The *C. elegans* neural editome reveals an ADAR target mRNA required for proper chemotaxis. elife. 2017;6:e28625 10.7554/eLife.28625 PMC5644944 28925356

[51] Dehmelt L, Halpain S. The MAP2/tau family of microtubule-associated proteins. Genome Biol. 2005;6:204 15642108 10.1186/gb-2004-6-1-204 PMC549057

[52] Del’Guidice T, Latapy C, Rampino A et al. FXR1P is a GSK3beta substrate regulating mood and emotion processing. Proc Natl Acad Sci USA. 2015;112:E4610–9 26240334 10.1073/pnas.1506491112 PMC4547302

[53] Desterro JM, Keegan LP, Lafarga M et al. Dynamic association of RNA-editing enzymes with the nucleolus. J Cell Sci. 2003;116:1805–18 12665561 10.1242/jcs.00371

[54] Dolzhanskaya N, Merz G, Aletta JM et al. Methylation regulates the intracellular protein–protein and protein–RNA interactions of FMRP. J Cell Sci. 2006;119:1933–46 16636078 10.1242/jcs.02882

[55] Donnelly CJ, Park M, Spillane M et al. Axonally synthesized-actin and GAP-43 proteins support distinct modes of axonal growth. J Neurosci. 2013;33:3311–22 23426659 10.1523/JNEUROSCI.1722-12.2013 PMC3711152

[56] Donnelly CJ, Willis DE, Xu M et al. Limited availability of ZBP1 restricts axonal mRNA localization and nerve regeneration capacity. EMBO J. 2011;30:4665–77 21964071 10.1038/emboj.2011.347 PMC3243598

[57] Dorsey SG, Renn CL, Carim-Todd L et al. *In vivo* restoration of physiological levels of truncated TrkB.T1 receptor rescues neuronal cell death in a trisomic mouse model. Neuron. 2006;51:21–8 16815329 10.1016/j.neuron.2006.06.009

[58] Dotti CG, Sullivan CA, Banker GA. The establishment of polarity by hippocampal neurons in culture. J Neurosci. 1988;8:1454–68 3282038 10.1523/JNEUROSCI.08-04-01454.1988 PMC6569279

[59] Dovey OM, Foster CT, Cowley SM. Histone deacetylase 1 (HDAC1), but not HDAC2, controls embryonic stem cell differentiation. Proc Natl Acad Sci USA. 2010;107:8242–7 20404188 10.1073/pnas.1000478107 PMC2889513

[60] Dubnau J, Chiang AS, Grady L et al. The staufen/pumilio pathway is involved in *Drosophila* long-term memory. Curr Biol. 2003;13:286–96 12593794 10.1016/s0960-9822(03)00064-2

[61] Dwivedi Y, Rizavi HS, Conley RR et al. Altered gene expression of brain-derived neurotrophic factor and receptor tyrosine kinase B in postmortem brain of suicide subjects. Arch Gen Psychiatry. 2003;60:804–15 12912764 10.1001/archpsyc.60.8.804

[62] Ebert DH, Greenberg ME. Activity-dependent neuronal signalling and autism spectrum disorder. Nature. 2013;493:327–37 23325215 10.1038/nature11860 PMC3576027

[63] Eckmann CR, Kraemer B, Wickens M et al. GLD-3, a bicaudal-C homolog that inhibits FBF to control germline sex determination in *C. elegans*. Dev Cell. 2002;3:697–710 12431376 10.1016/s1534-5807(02)00322-2

[64] Edbauer D, Neilson JR, Foster KA et al. Regulation of synaptic structure and function by FMRP-associated microRNAs miR-125b and miR-132. Neuron. 2010;65:373–84 20159450 10.1016/j.neuron.2010.01.005 PMC5018398

[65] Eisenberg E, Levanon EY. A-to-I RNA editing—immune protector and transcriptome diversifier. Nat Rev Genet. 2018;19:473–90 29692414 10.1038/s41576-018-0006-1

[66] Eom T, Antar LN, Singer RH et al. Localization of a beta-actin messenger ribonucleoprotein complex with zipcode-binding protein modulates the density of dendritic filopodia and filopodial synapses. J Neurosci. 2003;23:10433–44 14614102 10.1523/JNEUROSCI.23-32-10433.2003 PMC6741001

[67] Ernst C, Deleva V, Deng X et al. Alternative splicing, methylation state, and expression profile of tropomyosin-related kinase B in the frontal cortex of suicide completers. Arch Gen Psychiatry. 2009;66:22–32 19124685 10.1001/archpsyc.66.1.22

[68] Faghihi MA, Modarresi F, Khalil AM et al. Expression of a noncoding RNA is elevated in Alzheimer’s disease and drives rapid feed-forward regulation of beta-secretase. Nat Med. 2008;14:723–30 18587408 10.1038/nm1784 PMC2826895

[69] Faghihi MA, Zhang M, Huang J et al. Evidence for natural antisense transcript-mediated inhibition of microRNA function. Genome Biol. 2010;11:R56 20507594 10.1186/gb-2010-11-5-r56 PMC2898074

[70] Fallini C, Donlin-Asp PG, Rouanet JP et al. Deficiency of the survival of motor neuron protein impairs mRNA localization and local translation in the growth cone of motor neurons. J Neurosci. 2016;36:3811–20 27030765 10.1523/JNEUROSCI.2396-15.2016 PMC4812137

[71] Farina KL, Huttelmaier S, Musunuru K et al. Two ZBP1 KH domains facilitate beta-actin mRNA localization, granule formation, and cytoskeletal attachment. J Cell Biol. 2003;160:77–87 12507992 10.1083/jcb.200206003 PMC2172732

[72] Fatemi SH, Folsom TD. Dysregulation of fragile x mental retardation protein and metabotropic glutamate receptor 5 in superior frontal cortex of individuals with autism: a postmortem brain study. Mol Autism. 2011;2:6 21548960 10.1186/2040-2392-2-6 PMC3488976

[73] Fenner ME, Achim CL, Fenner BM. Expression of full-length and truncated trkB in human striatum and substantia nigra neurons: implications for Parkinson’s disease. J Mol Histol. 2014;45:349–61 24374887 10.1007/s10735-013-9562-z

[74] Fernandopulle MS, Lippincott-Schwartz J, Ward ME. RNA transport and local translation in neurodevelopmental and neurodegenerative disease. Nat Neurosci. 2021;24:622–32 33510479 10.1038/s41593-020-00785-2 PMC8860725

[75] Ferrer I, Marin C, Rey MJ et al. BDNF and full-length and truncated TrkB expression in Alzheimer disease. Implications in therapeutic strategies. J Neuropathol Exp Neurol. 1999;58:729–39 10411343 10.1097/00005072-199907000-00007

[76] Filippini A, Bonini D, Lacoux C et al. Absence of the fragile X mental retardation protein results in defects of RNA editing of neuronal mRNAs in mouse. RNA Biol. 2017;14:1580–91 28640668 10.1080/15476286.2017.1338232 PMC5785225

[77] Finkel RS, Chiriboga CA, Vajsar J et al. Treatment of infantile-onset spinal muscular atrophy with nusinersen: a phase 2, open-label, dose-escalation study. Lancet. 2016;388:3017–26 27939059 10.1016/S0140-6736(16)31408-8

[78] Fogel BL, Wexler E, Wahnich A et al. RBFOX1 regulates both splicing and transcriptional networks in human neuronal development. Hum Mol Genet. 2012;21:4171–86 22730494 10.1093/hmg/dds240 PMC3441119

[79] Freund EC, Sapiro AL, Li Q et al. Unbiased identification of *trans* regulators of ADAR and A-to-I RNA editing. Cell Rep. 2020;31:107656 32433965 10.1016/j.celrep.2020.107656 PMC7306178

[80] Fu XD, Ares MJ. Context-dependent control of alternative splicing by RNA-binding proteins. Nat Rev Genet. 2014;15:689–701 25112293 10.1038/nrg3778 PMC4440546

[81] Fujino T, Leslie JH, Eavri R et al. CPG15 regulates synapse stability in the developing and adult brain. Genes Dev. 2011;25:2674–85 22190461 10.1101/gad.176172.111 PMC3248687

[82] Gabel HW, Kinde B, Stroud H et al. Disruption of DNA-methylation-dependent long gene repression in Rett syndrome. Nature. 2015;522:89–93 25762136 10.1038/nature14319 PMC4480648

[83] Galgano A, Forrer M, Jaskiewicz L et al. Comparative analysis of mRNA targets for human PUF-family proteins suggests extensive interaction with the miRNA regulatory system. PLoS One. 2008;3:e3164 18776931 10.1371/journal.pone.0003164 PMC2522278

[84] Gandal MJ, Zhang P, Hadjimichael E et al. Transcriptome-wide isoform-level dysregulation in ASD, schizophrenia, and bipolar disorder. Science. 2018;362:eaat8127 10.1126/science.aat8127 PMC6443102 30545856

[85] Gehman LT, Meera P, Stoilov P et al. The splicing regulator Rbfox2 is required for both cerebellar development and mature motor function. Genes Dev. 2012;26:445–60 22357600 10.1101/gad.182477.111 PMC3305983

[86] Gehman LT, Stoilov P, Maguire J et al. The splicing regulator Rbfox1 (A2BP1) controls neuronal excitation in the mammalian brain. Nat Genet. 2011;43:706–11 21623373 10.1038/ng.841 PMC3125461

[87] Gupta S, Ellis SE, Ashar FN et al. Transcriptome analysis reveals dysregulation of innate immune response genes and neuronal activity-dependent genes in autism. Nat Commun. 2014;5:5748 25494366 10.1038/ncomms6748 PMC4270294

[88] Hagerman R, Hoem G, Hagerman P. Fragile X and autism: intertwined at the molecular level leading to targeted treatments. Mol Autism. 2010;1:12 20858229 10.1186/2040-2392-1-12 PMC2954865

[89] Han P, Chang CP. Long non-coding RNA and chromatin remodeling. RNA Biol. 2015;12:1094–8 26177256 10.1080/15476286.2015.1063770 PMC4829272

[90] Haque N, Ouda R, Chen C et al. ZFR coordinates crosstalk between RNA decay and transcription in innate immunity. Nat Commun. 2018;9:1145 29559679 10.1038/s41467-018-03326-5 PMC5861047

[91] Hedstrom KL, Ogawa Y, Rasband MN. AnkyrinG is required for maintenance of the axon initial segment and neuronal polarity. J Cell Biol. 2008;183:635–40 19001126 10.1083/jcb.200806112 PMC2582894

[92] Heidenreich KA, Linseman DA. Myocyte enhancer factor-2 transcription factors in neuronal differentiation and survival. Mol Neurobiol. 2004;29:155–66 15126683 10.1385/MN:29:2:155

[93] Hirokawa N. mRNA transport in dendrites: RNA granules, motors, and tracks. J Neurosci. 2006;26:7139–42 16822968 10.1523/JNEUROSCI.1821-06.2006 PMC6673940

[94] Hou L, Antion MD, Hu D et al. Dynamic translational and proteasomal regulation of fragile X mental retardation protein controls mGluR-dependent long-term depression. Neuron. 2006;51:441–54 16908410 10.1016/j.neuron.2006.07.005

[95] Hsieh J, Nakashima K, Kuwabara T et al. Histone deacetylase inhibition-mediated neuronal differentiation of multipotent adult neural progenitor cells. Proc Natl Acad Sci USA. 2004;101:16659–64 15537713 10.1073/pnas.0407643101 PMC527137

[96] Hu J, Qian H, Xue Y et al. PTB/nPTB: master regulators of neuronal fate in mammals. Biophys Rep. 2018;4:204–14 30310857 10.1007/s41048-018-0066-y PMC6153489

[97] Hua Y, Sahashi K, Rigo F et al. Peripheral SMN restoration is essential for long-term rescue of a severe spinal muscular atrophy mouse model. Nature. 2011;478:123–6 21979052 10.1038/nature10485 PMC3191865

[98] Huang YS, Jung MY, Sarkissian M et al. N-methyl-D-aspartate receptor signaling results in Aurora kinase-catalyzed CPEB phosphorylation and alpha CaMKII mRNA polyadenylation at synapses. EMBO J. 2002;21:2139–48 11980711 10.1093/emboj/21.9.2139 PMC125376

[99] Hughes TA. Regulation of gene expression by alternative untranslated regions. Trends Genet. 2006;22:119–22 16430990 10.1016/j.tig.2006.01.001

[100] Huttelmaier S, Zenklusen D, Lederer M et al. Spatial regulation of beta-actin translation by Src-dependent phosphorylation of ZBP1. Nature. 2005;438:512–5 16306994 10.1038/nature04115

[101] Iijima T, Yoshimura T. A perspective on the role of dynamic alternative RNA splicing in the development, specification, and function of axon initial segment. Front Mol Neurosci. 2019;12:295 31866821 10.3389/fnmol.2019.00295 PMC6906172

[102] Iossifov I, Ronemus M, Levy D et al. *De novo* gene disruptions in children on the autistic spectrum. Neuron. 2012;74:285–99 22542183 10.1016/j.neuron.2012.04.009 PMC3619976

[103] Ishizuka A, Siomi MC, Siomi H. A *Drosophila* fragile X protein interacts with components of RNAi and ribosomal proteins. Genes Dev. 2002;16:2497–508 12368261 10.1101/gad.1022002 PMC187455

[104] Jacko M, Weyn-Vanhentenryck SM, Smerdon JW et al. Rbfox splicing factors promote neuronal maturation and axon initial segment assembly. Neuron. 2018;97:853–68 29398366 10.1016/j.neuron.2018.01.020 PMC5823762

[105] Jansen A, Dieleman GC, Smit AB et al. Gene-set analysis shows association between FMRP targets and autism spectrum disorder. Eur J Hum Genet. 2017;25:863–8 28422133 10.1038/ejhg.2017.55 PMC5520067

[106] Jepson JEC, Savva YA, Yokose C et al. Engineered alterations in RNA editing modulate complex behavior in *Drosophila*: regulatory diversity of adenosine deaminase acting on RNA (ADAR) targets. J Biol Chem. 2011;286:8325–37 21078670 10.1074/jbc.M110.186817 PMC3048717

[107] Jin Y, Suzuki H, Maegawa S et al. A vertebrate RNA-binding protein Fox-1 regulates tissue-specific splicing via the pentanucleotide GCAUG. EMBO J. 2003;22:905–12 12574126 10.1093/emboj/cdg089 PMC145449

[108] Kaye JA, Rose NC, Goldsworthy B et al. A 3′UTR pumilio-binding element directs translational activation in olfactory sensory neurons. Neuron. 2009;61:57–70 19146813 10.1016/j.neuron.2008.11.012 PMC4274156

[109] Kiebler MA, Bassell GJ. Neuronal RNA granules: movers and makers. Neuron. 2006;51:685–90 16982415 10.1016/j.neuron.2006.08.021

[110] Knowles RB, Sabry JH, Martone ME et al. Translocation of RNA granules in living neurons. J Neurosci. 1996;16:7812–20 8987809 10.1523/JNEUROSCI.16-24-07812.1996 PMC6579227

[111] Kobayashi K, Kuroda S, Fukata M et al. p140Sra-1 (specifically Rac1-associated protein) is a novel specific target for Rac1 small GTPase. J Biol Chem. 1998;273:291–5 9417078 10.1074/jbc.273.1.291

[112] Kosik KS. The neuronal microRNA system. Nat Rev Neurosci. 2006;7:911–20 17115073 10.1038/nrn2037

[113] Krichevsky AM, Kosik KS. Neuronal RNA granules. Neuron. 2001;32:683–96 11719208 10.1016/s0896-6273(01)00508-6

[114] Kuroyanagi H. Fox-1 family of RNA-binding proteins. Cell Mol Life Sci. 2009;66:3895–907 19688295 10.1007/s00018-009-0120-5 PMC2777236

[115] L’Etoile ND, Coburn CM, Eastham J et al. The cyclic GMP-dependent protein kinase EGL-4 regulates olfactory adaptation in *C. elegans*. Neuron. 2002;36:1079–89 12495623 10.1016/s0896-6273(02)01066-8

[116] Lau AG, Irier HA, Gu J et al. Distinct 3′UTRs differentially regulate activity-dependent translation of brain-derived neurotrophic factor (BDNF). Proc Natl Acad Sci USA. 2010;107:15945–50 20733072 10.1073/pnas.1002929107 PMC2936648

[117] Lee JA, Damianov A, Lin CH et al. Cytoplasmic Rbfox1 regulates the expression of synaptic and autism-related genes. Neuron. 2016;89:113–28 26687839 10.1016/j.neuron.2015.11.025 PMC4858412

[118] Lee MH, Hook B, Pan G et al. Conserved regulation of MAP kinase expression by PUF RNA-binding proteins. PLoS Genet. 2007;3:e233 18166083 10.1371/journal.pgen.0030233 PMC2323325

[119] Lejeune F, Maquat LE. Mechanistic links between nonsense-mediated mRNA decay and pre-mRNA splicing in mammalian cells. Curr Opin Cell Biol. 2005;17:309–15 15901502 10.1016/j.ceb.2005.03.002

[120] Lennox AL, Mao H, Silver DL. RNA on the brain: emerging layers of post-transcriptional regulation in cerebral cortex development. Wiley Interdiscip Rev Dev Biol. 2018;7:10.1002/wdev.290 10.1002/wdev.290 PMC5746464 28837264

[121] Leppek K, Das R, Barna M. Functional 5′ UTR mRNA structures in eukaryotic translation regulation and how to find them. Nat Rev Mol Cell Biol. 2018;19:158–74 29165424 10.1038/nrm.2017.103 PMC5820134

[122] Leung KM, van Horck FP, Lin AC et al. Asymmetrical beta-actin mRNA translation in growth cones mediates attractive turning to netrin-1. Nat Neurosci. 2006;9:1247–56 16980963 10.1038/nn1775 PMC1997306

[123] Levanon EY, Eisenberg E, Yelin R et al. Systematic identification of abundant A-to-I editing sites in the human transcriptome. Nat Biotechnol. 2004;22:1001–5 15258596 10.1038/nbt996

[124] Lewis BP, Green RE, Brenner SE. Evidence for the widespread coupling of alternative splicing and nonsense-mediated mRNA decay in humans. Proc Natl Acad Sci USA. 2003;100:189–92 12502788 10.1073/pnas.0136770100 PMC140922

[125] Li L, Zhuang Y, Zhao X et al. Long non-coding RNA in neuronal development and neurological disorders. Front Genet. 2018;9:744 30728830 10.3389/fgene.2018.00744 PMC6351443

[126] Liao YC, Fernandopulle MS, Wang G et al. RNA granules hitchhike on lysosomes for long-distance transport, using Annexin A11 as a molecular tether. Cell. 2019;179:147–64 31539493 10.1016/j.cell.2019.08.050 PMC6890474

[127] Licatalosi DD, Mele A, Fak JJ et al. HITS-CLIP yields genome-wide insights into brain alternative RNA processing. Nature. 2008;456:464–9 18978773 10.1038/nature07488 PMC2597294

[128] Lin CH, Patton JG. Regulation of alternative 3′ splice site selection by constitutive splicing factors. RNA. 1995;1:234–45 7489496 PMC1369077

[129] Lin L, Goke J, Cukuroglu E et al. Molecular features underlying neurodegeneration identified through *in vitro* modeling of genetically diverse Parkinson’s disease patients. Cell Rep. 2016;15:2411–26 27264186 10.1016/j.celrep.2016.05.022

[130] Lin Y, Protter DS, Rosen MK et al. Formation and maturation of phase-separated liquid droplets by RNA-binding proteins. Mol Cell. 2015;60:208–19 26412307 10.1016/j.molcel.2015.08.018 PMC4609299

[131] Lipscombe D, Lopez Soto EJ. Alternative splicing of neuronal genes: new mechanisms and new therapies. Curr Opin Neurobiol. 2019;57:26–31 30703685 10.1016/j.conb.2018.12.013 PMC6629480

[132] Lopez Soto EJ, Gandal MJ, Gonatopoulos-Pournatzis T et al. Mechanisms of neuronal alternative splicing and strategies for therapeutic interventions. J Neurosci. 2019;39:8193–9 31619487 10.1523/JNEUROSCI.1149-19.2019 PMC6794923

[133] Lopez-Erauskin J, Tadokoro T, Baughn MW et al. ALS/FTD-linked mutation in FUS suppresses intra-axonal protein synthesis and drives disease without nuclear loss-of-function of FUS. Neuron. 2018;100:816–30 30344044 10.1016/j.neuron.2018.09.044 PMC6277851

[134] Lu B, Nagappan G, Guan X et al. BDNF-based synaptic repair as a disease-modifying strategy for neurodegenerative diseases. Nat Rev Neurosci. 2013;14:401–16 23674053 10.1038/nrn3505

[135] Maday S, Twelvetrees AE, Moughamian AJ et al. Axonal transport: cargo-specific mechanisms of motility and regulation. Neuron. 2014;84:292–309 25374356 10.1016/j.neuron.2014.10.019 PMC4269290

[136] Makeyev EV, Zhang J, Carrasco MA et al. The microRNA miR-124 promotes neuronal differentiation by triggering brain-specific alternative pre-mRNA splicing. Mol Cell. 2007;27:435–48 17679093 10.1016/j.molcel.2007.07.015 PMC3139456

[137] Maldonado C, Alicea D, Gonzalez M et al. Adar is essential for optimal presynaptic function. Mol Cell Neurosci. 2013;52:173–80 23127996 10.1016/j.mcn.2012.10.009 PMC3613243

[138] Mao Z, Bonni A, Xia F et al. Neuronal activity-dependent cell survival mediated by transcription factor MEF2. Science. 1999;286:785–90 10531066 10.1126/science.286.5440.785

[139] Markovtsov V, Nikolic JM, Goldman JA et al. Cooperative assembly of an hnRNP complex induced by a tissue-specific homolog of polypyrimidine tract binding protein. Mol Cell Biol. 2000;20:7463–79 11003644 10.1128/mcb.20.20.7463-7479.2000 PMC86300

[140] Martin CL, Duvall JA, Ilkin Y et al. Cytogenetic and molecular characterization of A2BP1/FOX1 as a candidate gene for autism. Am J Med Genet B Neuropsychiatr Genet. 2007;144B:869–76 17503474 10.1002/ajmg.b.30530

[141] Martinez JC, Randolph LK, Iascone DM et al. Pum2 shapes the transcriptome in developing axons through retention of target mRNAs in the cell body. Neuron. 2019;104:931–46 31606248 10.1016/j.neuron.2019.08.035 PMC6895424

[142] Matlin AJ, Clark F, Smith CW. Understanding alternative splicing: towards a cellular code. Nat Rev Mol Cell Biol. 2005;6:386–98 15956978 10.1038/nrm1645

[143] Mauger O, Scheiffele P. Beyond proteome diversity: alternative splicing as a regulator of neuronal transcript dynamics. Curr Opin Neurobiol. 2017;45:162–8 28609697 10.1016/j.conb.2017.05.012 PMC6689270

[144] Medioni C, Ramialison M, Ephrussi A et al. Imp promotes axonal remodeling by regulating profilin mRNA during brain development. Curr Biol. 2014;24:793–800 24656828 10.1016/j.cub.2014.02.038

[145] Mee CJ, Pym EC, Moffat KG et al. Regulation of neuronal excitability through pumilio-dependent control of a sodium channel gene. J Neurosci. 2004;24:8695–703 15470135 10.1523/JNEUROSCI.2282-04.2004 PMC6729971

[146] Menon KP, Sanyal S, Habara Y et al. The translational repressor pumilio regulates presynaptic morphology and controls postsynaptic accumulation of translation factor eIF-4E. Neuron. 2004;44:663–76 15541314 10.1016/j.neuron.2004.10.028

[147] Merianda TT, Gomes C, Yoo S et al. Axonal localization of neuritin/CPG15 mRNA in neuronal populations through distinct 5′ and 3′ UTR elements. J Neurosci. 2013;33:13735–42 23966695 10.1523/JNEUROSCI.0962-13.2013 PMC3755718

[148] Miao H, Wang L, Zhan H et al. A long noncoding RNA distributed in both nucleus and cytoplasm operates in the PYCARD-regulated apoptosis by coordinating the epigenetic and translational regulation. PLoS Genet. 2019;15:e1008144 31086376 10.1371/journal.pgen.1008144 PMC6534332

[149] Minichiello L. TrkB signalling pathways in LTP and learning. Nat Rev Neurosci. 2009;10:850–60 19927149 10.1038/nrn2738

[150] Mobarak CD, Anderson KD, Morin M et al. The RNA-binding protein HuD is required for GAP-43 mRNA stability, GAP-43 gene expression, and PKC-dependent neurite outgrowth in PC12 cells. Mol Biol Cell. 2000;11:3191–203 10982410 10.1091/mbc.11.9.3191 PMC14985

[151] Modic M, Ule J, Sibley CR. CLIPing the brain: studies of protein-RNA interactions important for neurodegenerative disorders. Mol Cell Neurosci. 2013;56:429–35 23583633 10.1016/j.mcn.2013.04.002 PMC3793874

[152] Moore FL, Jaruzelska J, Fox MS et al. Human Pumilio-2 is expressed in embryonic stem cells and germ cells and interacts with DAZ (deleted in AZoospermia) and DAZ-like proteins. Proc Natl Acad Sci USA. 2003;100:538–43 12511597 10.1073/pnas.0234478100 PMC141031

[153] Muddashetty RS, Nalavadi VC, Gross C et al. Reversible inhibition of PSD-95 mRNA translation by miR-125a, FMRP phosphorylation, and mGluR signaling. Mol Cell. 2011;42:673–88 21658607 10.1016/j.molcel.2011.05.006 PMC3115785

[154] Naeve GS, Ramakrishnan M, Kramer R et al. Neuritin: a gene induced by neural activity and neurotrophins that promotes neuritogenesis. Proc Natl Acad Sci USA. 1997;94:2648–53 9122250 10.1073/pnas.94.6.2648 PMC20143

[155] Napoli I, Mercaldo V, Boyl PP et al. The fragile X syndrome protein represses activity-dependent translation through CYFIP1, a new 4E-BP. Cell. 2008;134:1042–54 18805096 10.1016/j.cell.2008.07.031

[156] Narayanan U, Nalavadi V, Nakamoto M et al. FMRP phosphorylation reveals an immediate-early signaling pathway triggered by group I mGluR and mediated by PP2A. J Neurosci. 2007;27:14349–57 18160642 10.1523/JNEUROSCI.2969-07.2007 PMC6673448

[157] Narayanan U, Nalavadi V, Nakamoto M et al. S6K1 phosphorylates and regulates fragile X mental retardation protein (FMRP) with the neuronal protein synthesis-dependent mammalian target of rapamycin (mTOR) signaling cascade. J Biol Chem. 2008;283:18478–82 18474609 10.1074/jbc.C800055200 PMC2441545

[158] Nedivi E, Fieldust S, Theill LE et al. A set of genes expressed in response to light in the adult cerebral cortex and regulated during development. Proc Natl Acad Sci USA. 1996;93:2048–53 8700883 10.1073/pnas.93.5.2048 PMC39907

[159] Nicastro G, Candel AM, Uhl M et al. Mechanism of β-actin mRNA recognition by ZBP1. Cell Rep. 2017;18:1187–99 28147274 10.1016/j.celrep.2016.12.091 PMC5300891

[160] Nishikura K. Functions and regulation of RNA editing by ADAR deaminases. Annu Rev Biochem. 2010;79:321–49 20192758 10.1146/annurev-biochem-060208-105251 PMC2953425

[161] Nishikura K. A-to-I editing of coding and non-coding RNAs by ADARs. Nat Rev Mol Cell Biol. 2016;17:83–96 26648264 10.1038/nrm.2015.4 PMC4824625

[162] Nishino J, Kim S, Zhu Y et al. A network of heterochronic genes including Imp1 regulates temporal changes in stem cell properties. elife. 2013;2:e00924 24192035 10.7554/eLife.00924 PMC3817382

[163] Oakes E, Anderson A, Cohen-Gadol A et al. Adenosine deaminase that acts on RNA 3 (ADAR3) binding to glutamate receptor subunit B pre-mRNA inhibits RNA editing in glioblastoma. J Biol Chem. 2017;292:4326–35 28167531 10.1074/jbc.M117.779868 PMC5354488

[164] Oberstrass FC, Auweter SD, Erat M et al. Structure of PTB bound to RNA: specific binding and implications for splicing regulation. Science. 2005;309:2054–7 16179478 10.1126/science.1114066

[165] Oe S, Yoneda Y. Cytoplasmic polyadenylation element-like sequences are involved in dendritic targeting of BDNF mRNA in hippocampal neurons. FEBS Lett. 2010;584:3424–30 20603120 10.1016/j.febslet.2010.06.040

[166] Okamura K, Ishizuka A, Siomi H et al. Distinct roles for Argonaute proteins in small RNA-directed RNA cleavage pathways. Genes Dev. 2004;18:1655–66 15231716 10.1101/gad.1210204 PMC478188

[167] Panja D, Kenney JW, D’Andrea L et al. Two-stage translational control of dentate gyrus LTP consolidation is mediated by sustained BDNF-TrkB signaling to MNK. Cell Rep. 2014;9:1430–45 25453757 10.1016/j.celrep.2014.10.016

[168] Parikshak NN, Luo R, Zhang A et al. Integrative functional genomic analyses implicate specific molecular pathways and circuits in autism. Cell. 2013;155:1008–21 24267887 10.1016/j.cell.2013.10.031 PMC3934107

[169] Park H, Poo MM. Neurotrophin regulation of neural circuit development and function. Nat Rev Neurosci. 2013;14:7–23 23254191 10.1038/nrn3379

[170] Parvin S, Takeda R, Sugiura Y et al. Fragile X mental retardation protein regulates accumulation of the active zone protein Munc18-1 in presynapses via local translation in axons during synaptogenesis. Neurosci Res. 2019;146:36–47 30240639 10.1016/j.neures.2018.09.013

[171] Patel VL, Mitra S, Harris R et al. Spatial arrangement of an RNA zipcode identifies mRNAs under post-transcriptional control. Genes Dev. 2012;26:43–53 22215810 10.1101/gad.177428.111 PMC3258965

[172] Pattabiraman PP, Tropea D, Chiaruttini C et al. Neuronal activity regulates the developmental expression and subcellular localization of cortical BDNF mRNA isoforms *in vivo*. Mol Cell Neurosci. 2005;28:556–70 15737745 10.1016/j.mcn.2004.11.010

[173] Pelka GJ, Watson CM, Christodoulou J et al. Distinct expression profiles of Mecp2 transcripts with different lengths of 3′UTR in the brain and visceral organs during mouse development. Genomics. 2005;85:441–52 15780747 10.1016/j.ygeno.2004.12.002

[174] Pinto D, Delaby E, Merico D et al. Convergence of genes and cellular pathways dysregulated in autism spectrum disorders. Am J Hum Genet. 2014;94:677–94 24768552 10.1016/j.ajhg.2014.03.018 PMC4067558

[175] Plante I, Davidovic L, Ouellet DL et al. Dicer-derived microRNAs are utilized by the fragile X mental retardation protein for assembly on target RNAs. J Biomed Biotechnol. 2006;2006:64347–12 17057366 10.1155/JBB/2006/64347 PMC1698263

[176] Polydorides AD, Okano HJ, Yang YY et al. A brain-enriched polypyrimidine tract-binding protein antagonizes the ability of Nova to regulate neuron-specific alternative splicing. Proc Natl Acad Sci USA. 2000;97:6350–5 10829067 10.1073/pnas.110128397 PMC18606

[177] Ponthier JL, Schluepen C, Chen W et al. Fox-2 splicing factor binds to a conserved intron motif to promote inclusion of protein 4.1R alternative exon 16. J Biol Chem. 2006;281:12468–74 16537540 10.1074/jbc.M511556200

[178] Pushpalatha KV, Besse F. Local translation in axons: when membraneless RNP granules meet membrane-bound organelles. Front Mol Biosci. 2019;6:129 31824961 10.3389/fmolb.2019.00129 PMC6882739

[179] Quinones-Valdez G, Tran SS, Jun HI et al. Regulation of RNA editing by RNA-binding proteins in human cells. Commun Biol. 2019;2:19 30652130 10.1038/s42003-018-0271-8 PMC6331435

[180] Ramos A, Hollingworth D, Pastore A. G-quartet-dependent recognition between the FMRP RGG box and RNA. RNA. 2003;9:1198–207 13130134 10.1261/rna.5960503 PMC1370484

[181] Rasband MN. The axon initial segment and the maintenance of neuronal polarity. Nat Rev Neurosci. 2010;11:552–62 20631711 10.1038/nrn2852

[182] Richter JD, Sonenberg N. Regulation of cap-dependent translation by eIF4E inhibitory proteins. Nature. 2005;433:477–80 15690031 10.1038/nature03205

[183] Robinson JE, Paluch J, Dickman DK et al. ADAR-mediated RNA editing suppresses sleep by acting as a brake on glutamatergic synaptic plasticity. Nat Commun. 2016;7:10512 26813350 10.1038/ncomms10512 PMC4737855

[184] Rosenthal JJ, Seeburg PH. A-to-I RNA editing: effects on proteins key to neural excitability. Neuron. 2012;74:432–9 22578495 10.1016/j.neuron.2012.04.010 PMC3724421

[185] Salvatori B, Biscarini S, Morlando M. Non-coding RNAs in nervous system development and disease. Front Cell Dev Biol. 2020;8:273 32435641 10.3389/fcell.2020.00273 PMC7218086

[186] Sansam CL, Wells KS, Emeson RB. Modulation of RNA editing by functional nucleolar sequestration of ADAR2. Proc Natl Acad Sci USA. 2003;100:14018–23 14612560 10.1073/pnas.2336131100 PMC283538

[187] Sapiro AL, Freund EC, Restrepo L et al. Zinc finger RNA-binding protein Zn72D regulates ADAR-mediated RNA editing in neurons. Cell Rep. 2020;31:107654 32433963 10.1016/j.celrep.2020.107654 PMC7306179

[188] Sasaki Y, Welshhans K, Wen Z et al. Phosphorylation of zipcode binding protein 1 is required for brain-derived neurotrophic factor signaling of local beta-actin synthesis and growth cone turning. J Neurosci. 2010;30:9349–58 20631164 10.1523/JNEUROSCI.0499-10.2010 PMC2908896

[189] Schenck A, Bardoni B, Langmann C et al. CYFIP/Sra-1 controls neuronal connectivity in *Drosophila* and links the Rac1 GTPase pathway to the fragile X protein. Neuron. 2003;38:887–98 12818175 10.1016/s0896-6273(03)00354-4

[190] Sebat J, Lakshmi B, Malhotra D et al. Strong association of de novo copy number mutations with autism. Science. 2007;316:445–9 17363630 10.1126/science.1138659 PMC2993504

[191] Severt WL, Biber TU, Wu X et al. The suppression of testis-brain RNA binding protein and kinesin heavy chain disrupts mRNA sorting in dendrites. J Cell Sci. 1999;112:3691–702 10523505 10.1242/jcs.112.21.3691

[192] Shalizi AK, Bonni A. Brawn for brains: the role of MEF2 proteins in the developing nervous system. Curr Top Dev Biol. 2005;69:239–66 16243602 10.1016/S0070-2153(05)69009-6

[193] Shamay-Ramot A, Khermesh K, Porath HT et al. Fmrp interacts with Adar and regulates RNA editing, synaptic density and locomotor activity in zebrafish. PLoS Genet. 2015;11:e1005702 26637167 10.1371/journal.pgen.1005702 PMC4670233

[194] Singh R, Valcarcel J, Green MR. Distinct binding specificities and functions of higher eukaryotic polypyrimidine tract-binding proteins. Science. 1995;268:1173–6 7761834 10.1126/science.7761834

[195] Sinha R, Kim YJ, Nomakuchi T et al. Antisense oligonucleotides correct the familial dysautonomia splicing defect in IKBKAP transgenic mice. Nucleic Acids Res. 2018;46:4833–44 29672717 10.1093/nar/gky249 PMC6007753

[196] Slotkin W, Nishikura K. Adenosine-to-inosine RNA editing and human disease. Genome Med. 2013;5:105 24289319 10.1186/gm508 PMC3979043

[197] Spassov DS, Jurecic R. The PUF family of RNA-binding proteins: does evolutionarily conserved structure equal conserved function? IUBMB Life. 2003;55:359–66 14584586 10.1080/15216540310001603093

[198] Spellman R, Smith CW. Novel modes of splicing repression by PTB. Trends Biochem Sci. 2006;31:73–6 16403634 10.1016/j.tibs.2005.12.003

[199] Statello L, Guo CJ, Chen LL et al. Gene regulation by long non-coding RNAs and its biological functions. Nat Rev Mol Cell Biol. 2021;22:96–118 33353982 10.1038/s41580-020-00315-9 PMC7754182

[200] Sugino K, Clark E, Schulmann A et al. Mapping the transcriptional diversity of genetically and anatomically defined cell populations in the mouse brain. elife. 2019;8:8 10.7554/eLife.38619 PMC6499542 30977723

[201] Sugino K, Hempel CM, Okaty BW et al. Cell-type-specific repression by methyl-CpG-binding protein 2 is biased toward long genes. J Neurosci. 2014;34:12877–83 25232122 10.1523/JNEUROSCI.2674-14.2014 PMC4166166

[202] Sutton MA, Schuman EM. Local translational control in dendrites and its role in long-term synaptic plasticity. J Neurobiol. 2005;64:116–31 15883999 10.1002/neu.20152

[203] Sutton MA, Schuman EM. Dendritic protein synthesis, synaptic plasticity, and memory. Cell. 2006;127:49–58 17018276 10.1016/j.cell.2006.09.014

[204] Tan MH, Li Q, Shanmugam R et al. Dynamic landscape and regulation of RNA editing in mammals. Nature. 2017;550:249–54 29022589 10.1038/nature24041 PMC5723435

[205] Tariq A, Garncarz W, Handl C et al. RNA-interacting proteins act as site-specific repressors of ADAR2-mediated RNA editing and fluctuate upon neuronal stimulation. Nucleic Acids Res. 2013;41:2581–93 23275536 10.1093/nar/gks1353 PMC3575830

[206] Tariq A, Jantsch MF. Transcript diversification in the nervous system: a to I RNA editing in CNS function and disease development. Front Neurosci. 2012;6:99 22787438 10.3389/fnins.2012.00099 PMC3391646

[207] Taylor AM, Berchtold NC, Perreau VM et al. Axonal mRNA in uninjured and regenerating cortical mammalian axons. J Neurosci. 2009;29:4697–707 19369540 10.1523/JNEUROSCI.6130-08.2009 PMC3632375

[208] Thoenen H. Neurotrophins and neuronal plasticity. Science. 1995;270:593–8 7570017 10.1126/science.270.5236.593

[209] Tichon A, Gil N, Lubelsky Y et al. A conserved abundant cytoplasmic long noncoding RNA modulates repression by pumilio proteins in human cells. Nat Commun. 2016;7:12209 27406171 10.1038/ncomms12209 PMC4947167

[210] Tomassoni-Ardori F, Fulgenzi G, Becker J et al. Rbfox1 up-regulation impairs BDNF-dependent hippocampal LTP by dysregulating TrkB isoform expression levels. elife. 2019;8:e49673 10.7554/eLife.49673 PMC6715404 31429825

[211] Tran SS, Jun HI, Bahn JH et al. Widespread RNA editing dysregulation in brains from autistic individuals. Nat Neurosci. 2019;22:25–36 30559470 10.1038/s41593-018-0287-x PMC6375307

[212] Traunmuller L, Bornmann C, Scheiffele P. Alternative splicing coupled nonsense-mediated decay generates neuronal cell type-specific expression of SLM proteins. J Neurosci. 2014;34:16755–61 25505328 10.1523/JNEUROSCI.3395-14.2014 PMC6608507

[213] Traunmuller L, Gomez AM, Nguyen TM et al. Control of neuronal synapse specification by a highly dedicated alternative splicing program. Science. 2016;352:982–6 27174676 10.1126/science.aaf2397

[214] Tsang B, Arsenault J, Vernon RM et al. Phosphoregulated FMRP phase separation models activity-dependent translation through bidirectional control of mRNA granule formation. Proc Natl Acad Sci USA. 2019;116:4218–27 30765518 10.1073/pnas.1814385116 PMC6410804

[215] Tushev G, Glock C, Heumuller M et al. Alternative 3′ UTRs modify the localization, regulatory potential, stability, and plasticity of mRNAs in neuronal compartments. Neuron. 2018;98:495–511 29656876 10.1016/j.neuron.2018.03.030

[216] Underwood JG, Boutz PL, Dougherty JD et al. Homologues of the *Caenorhabditis elegans* Fox-1 protein are neuronal splicing regulators in mammals. Mol Cell Biol. 2005;25:10005–16 16260614 10.1128/MCB.25.22.10005-10016.2005 PMC1280273

[217] Vernon RM, Chong PA, Tsang B et al. Pi-Pi contacts are an overlooked protein feature relevant to phase separation. elife. 2018;7:e31486 10.7554/eLife.31486 PMC5847340 29424691

[218] Vesely C, Jantsch MF. An I for an A: dynamic regulation of adenosine deamination-mediated RNA editing. Genes (Basel). 2021;12:12 10.3390/genes12071026 PMC8304401 34356042

[219] Vessey JP, Macchi P, Stein JM et al. A loss of function allele for murine Staufen1 leads to impairment of dendritic Staufen1-RNP delivery and dendritic spine morphogenesis. Proc Natl Acad Sci USA. 2008;105:16374–9 18922781 10.1073/pnas.0804583105 PMC2567905

[220] Vicario A, Colliva A, Ratti A et al. Dendritic targeting of short and long 3′ UTR BDNF mRNA is regulated by BDNF or NT-3 and distinct sets of RNA-binding proteins. Front Mol Neurosci. 2015;8:62 26578876 10.3389/fnmol.2015.00062 PMC4624863

[221] Vogt MA, Ehsaei Z, Knuckles P et al. TDP-43 induces p53-mediated cell death of cortical progenitors and immature neurons. Sci Rep. 2018;8:8097 29802307 10.1038/s41598-018-26397-2 PMC5970242

[222] Vuong CK, Wei W, Lee JA et al. Rbfox1 regulates synaptic transmission through the inhibitory neuron-specific vSNARE Vamp1. Neuron. 2018;98:127–41 29621484 10.1016/j.neuron.2018.03.008 PMC5890944

[223] Vuong JK, Lin CH, Zhang M et al. PTBP1 and PTBP2 serve both specific and redundant functions in neuronal pre-mRNA splicing. Cell Rep. 2016;17:2766–75 27926877 10.1016/j.celrep.2016.11.034 PMC5179036

[224] Wagner EJ, Garcia-Blanco MA. Polypyrimidine tract binding protein antagonizes exon definition. Mol Cell Biol. 2001;21:3281–8 11313454 10.1128/MCB.21.10.3281-3288.2001 PMC100250

[225] Wang L, Eckmann CR, Kadyk LC et al. A regulatory cytoplasmic poly(a) polymerase in *Caenorhabditis elegans*. Nature. 2002a;419:312–6 12239571 10.1038/nature01039

[226] Wang W, Wei Z, Li H. A change-point model for identifying 3′UTR switching by next-generation RNA sequencing. Bioinformatics. 2014;30:2162–70 24728858 10.1093/bioinformatics/btu189 PMC4103598

[227] Wang Y, Liu X, Biederer T et al. A family of RIM-binding proteins regulated by alternative splicing: implications for the genesis of synaptic active zones. Proc Natl Acad Sci USA. 2002b;99:14464–9 12391317 10.1073/pnas.182532999 PMC137906

[228] Washburn MC, Hundley HA. *Trans* and *cis* factors affecting A-to-I RNA editing efficiency of a noncoding editing target in *C. elegans*. RNA. 2016;22:722–8 26917557 10.1261/rna.055079.115 PMC4836646

[229] Washburn MC, Kakaradov B, Sundararaman B et al. The dsRBP and inactive editor ADR-1 utilizes dsRNA binding to regulate A-to-I RNA editing across the *C. elegans* transcriptome. Cell Rep. 2014;6:599–607 24508457 10.1016/j.celrep.2014.01.011 PMC3959997

[230] Wei PC, Chang AN, Kao J et al. Long neural genes harbor recurrent DNA break clusters in neural stem/progenitor cells. Cell. 2016;164:644–55 26871630 10.1016/j.cell.2015.12.039 PMC4752721

[231] Welshhans K, Bassell GJ. Netrin-1-induced local—actin synthesis and growth cone guidance requires zipcode binding protein 1. J Neurosci. 2011;31:9800–13 21734271 10.1523/JNEUROSCI.0166-11.2011 PMC3137872

[232] Westmark CJ, Malter JS. FMRP mediates mGluR5-dependent translation of amyloid precursor protein. PLoS Biol. 2007;5:e52 17298186 10.1371/journal.pbio.0050052 PMC1808499

[233] Weyn-Vanhentenryck SM, Mele A, Yan Q et al. HITS-CLIP and integrative modeling define the Rbfox splicing-regulatory network linked to brain development and autism. Cell Rep. 2014;6:1139–52 24613350 10.1016/j.celrep.2014.02.005 PMC3992522

[234] Wickens M, Bernstein DS, Kimble J et al. A PUF family portrait: 3′UTR regulation as a way of life. Trends Genet. 2002;18:150–7 11858839 10.1016/s0168-9525(01)02616-6

[235] Will TJ, Tushev G, Kochen L et al. Deep sequencing and high-resolution imaging reveal compartment-specific localization ofBdnfmRNA in hippocampal neurons. Sci Signal. 2013;6:rs16 24345682 10.1126/scisignal.2004520 PMC5321484

[236] Willis DE, van Niekerk EA, Sasaki Y et al. Extracellular stimuli specifically regulate localized levels of individual neuronal mRNAs. J Cell Biol. 2007;178:965–80 17785519 10.1083/jcb.200703209 PMC2064621

[237] Worringer KA, Panning B. Zinc finger protein Zn72D promotes productive splicing of the maleless transcript. Mol Cell Biol. 2007;27:8760–9 17923683 10.1128/MCB.01415-07 PMC2169391

[238] Wu B, Buxbaum AR, Katz ZB et al. Quantifying protein–mRNA interactions in single live cells. Cell. 2015;162:211–20 26140598 10.1016/j.cell.2015.05.054 PMC4491145

[239] Xia Z, Donehower LA, Cooper TA et al. Dynamic analyses of alternative polyadenylation from RNA-seq reveal a 3′-UTR landscape across seven tumour types. Nat Commun. 2014;5:5274 25409906 10.1038/ncomms6274 PMC4467577

[240] Xu B, Roos JL, Levy S et al. Strong association of *de novo* copy number mutations with sporadic schizophrenia. Nat Genet. 2008;40:880–5 18511947 10.1038/ng.162

[241] Xue Y, Ouyang K, Huang J et al. Direct conversion of fibroblasts to neurons by reprogramming PTB-regulated microRNA circuits. Cell. 2013;152:82–96 23313552 10.1016/j.cell.2012.11.045 PMC3552026

[242] Yan Q, Weyn-Vanhentenryck SM, Wu J et al. Systematic discovery of regulated and conserved alternative exons in the mammalian brain reveals NMD modulating chromatin regulators. Proc Natl Acad Sci USA. 2015;112:3445–50 25737549 10.1073/pnas.1502849112 PMC4371929

[243] Ye B, Petritsch C, Clark IE et al. Nanos and pumilio are essential for dendrite morphogenesis in *Drosophila* peripheral neurons. Curr Biol. 2004;14:314–21 14972682 10.1016/j.cub.2004.01.052

[244] Ye C, Long Y, Ji G et al. APAtrap: identification and quantification of alternative polyadenylation sites from RNA-seq data. Bioinformatics. 2018;34:1841–9 29360928 10.1093/bioinformatics/bty029

[245] Yoo AS, Sun AX, Li L et al. MicroRNA-mediated conversion of human fibroblasts to neurons. Nature. 2011;476:228–31 21753754 10.1038/nature10323 PMC3348862

[246] Yoo S, Kim HH, Kim P et al. A HuD-ZBP1 ribonucleoprotein complex localizes GAP-43 mRNA into axons through its 3′ untranslated region AU-rich regulatory element. J Neurochem. 2013;126:792–804 23586486 10.1111/jnc.12266 PMC3766383

[247] Yoon JH, Abdelmohsen K, Gorospe M. Posttranscriptional gene regulation by long noncoding RNA. J Mol Biol. 2013;425:3723–30 23178169 10.1016/j.jmb.2012.11.024 PMC3594629

[248] Zhang H, Lee JY, Tian B. Biased alternative polyadenylation in human tissues. Genome Biol. 2005;6:R100 16356263 10.1186/gb-2005-6-12-r100 PMC1414089

[249] Zhang M, Chen D, Xia J et al. Post-transcriptional regulation of mouse neurogenesis by pumilio proteins. Genes Dev. 2017;31:1354–69 28794184 10.1101/gad.298752.117 PMC5580656

[250] Zhang Y, O'Connor JP, Siomi MC et al. The fragile X mental retardation syndrome protein interacts with novel homologs FXR1 and FXR2. EMBO J. 1995;14:5358–66 7489725 10.1002/j.1460-2075.1995.tb00220.x PMC394645

[251] Zhao C, Takita J, Tanaka Y et al. Charcot-Marie-Tooth disease type 2A caused by mutation in a microtubule motor KIF1Bbeta. Cell. 2001;105:587–97 11389829 10.1016/s0092-8674(01)00363-4

[252] Zhu B, Ramachandran B, Gulick T. Alternative pre-mRNA splicing governs expression of a conserved acidic transactivation domain in myocyte enhancer factor 2 factors of striated muscle and brain. J Biol Chem. 2005;280:28749–60 15834131 10.1074/jbc.M502491200

[253] Zylka MJ, Simon JM, Philpot BD. Gene length matters in neurons. Neuron. 2015;86:353–5 25905808 10.1016/j.neuron.2015.03.059 PMC4584405

