# Supplementary material for: RNA and neuronal function: the importance of post-transcriptional regulation
Source: Oxf Open Neurosci. 2022 Jul 7;1:kvac011. doi: 10.1093/oons/kvac011 (PMC10913846; doi:10.1093/oons/kvac011)
Supplement: suppl_data_kvac011 [file suppl_data_kvac011.zip › reviewer comments for transparent peer review.docx]

**OXFNSC-2022-007 RNA and neuronal function: the importance of post-transcriptional regulation**

**Reviewer comments original submission**

Reviewer 1

This manuscript broadly reviews post-transcriptional regulation in neurons. Whilst it is generally well written, I have various suggestions below for improvements.

1. The title ‘RNA and neuronal function: the importance of post transcriptional regulation’ is extremely broad. It is very difficult for the authors to thoroughly review and discuss such a broad topic. As a result, their coverage of each component is somewhat patchy and incomplete. The authors should think about not only a more focused title, but also focusing the overall manuscript more to comprehensively cover one or more aspects in detail.

2. Considering the breadth of the topic, the three figures, whilst high quality, are insufficient. They do not adequately cover the range of molecular and cellular processes discussed. And Figure 3 focuses on GAP-43, so at least one more figure is needed that more generally covered intracellular mRNA trafficking and spatial control of translation.

3. Is translational control, not specifically mentioned in the Abstract, in the remit of this manuscript?

4. Considering the breadth of literature covered, it is surprising there are no tables. Perhaps one or more tables would help categorise and systematise, as well as integrate, the key studies in each aspect of the manuscript.

5. The authors focus on neurons (and focus is a good thing within the broad topic, as mentioned above) but they might want to mention in the Abstract (and/or Introduction) the topics they are excluding, including glial cells.

6. Are the authors distinguishing between developing and mature neurons? This could be made more explicit. And they should make it clear that neurons in vitro never fully mature, so that is a caveat of all such studies.

7. One novel and important aspect of such RNA regulation is the ‘repeatome’, that approximately half of the human genome consisting of repetitive sequences. Tandem repeats in particular are increasingly being implicated in a range of post-transcriptional molecular and cellular processes including those disrupted in tandem-repeat disorders such as fragile X syndrome (reviewed in Nat. Rev. Genet. 2018; 19:286-298).

8. What about short and long noncoding RNAs? Whilst miRNAs are covered, there are many other non-coding RNAs not discussed. If this is not in the scope of the review, the authors should explicitly state this up front.

Reviewer 2

The intracellular mechanisms involved in RNA post-transcriptional regulation are thoroughly described in this manuscript. However, there is little information on how these mechanisms connect to extracellular cues. When available, authors might include a layer connecting inputs derived from the extracellular space that determine or trigger the reviewed molecular mechanisms.

The manuscript contains numerous good examples that support RNA processing reviewed by authors. However, the three figures in the manuscript only depict a tiny fraction of those examples. I would suggest including a Table that summarizes the effects of RNA editing on the transcripts and its functional outcomes for brain or neuronal functions.

Lines 112-127 describe the SA of ERC1a and ERC1b. Please include a reference to Figure 1b to direct readers to the relevant part of the figure

Lines 128-136 describe how AS for neurexin is essential for synaptic functions. Please include a brief description of the functions of neuraxin and how such SA mechanisms provide a layer of regulation for synapses and plasticity.

Lines 164-166 state that Rbfox protein forms a multi-protein complex that includes hnRNP M, H, and C; Matrin3, NF110, NF45, and DDX5. Authors may want to include a brief description of these proteins as non-expert might not know the implications for these protein-protein interactions.

Lines 176-184 describe the AS of AnkG that Rbfox mediates. I would suggest expanding the authors' ideas on why the exclusion of the alternative exon is required to foster the interaction of AnkG with the actin cytoskeleton. In addition, please refers to the text Figure 1c to direct readers to the relevant part of the figure.

Indicate in the text where Figure 1d is cited. It is not apparent to readers (maybe lines 152-168?)

Lines 190-198 include a reference to figure 1a to direct readers to the relevant part of the figure.

Lines 247-249 indicate that ADAR suppression in Drosophila alters synaptic plasticity associated with modifications in glutamatergic signaling. Authors might explain the targets for the glutamatergic signaling associated with ADAR functions.

Lines 556-570 could be summarized and depicted in a Figure.

Lines 601-603 refer to anterograde and retrograde transport of RNA granules associated with membraned organelles. Authors may want to discuss which organelles are involved in such transport, including relevant references.

**Reviewer comments revised submission**

Reviewer 1

The manuscript has been revised and my comments adequately addressed.

Reviewer 2

The authors adequately addressed the concerns raised after the initial submission.
